# Comprehensive atmospheric modeling of reactive cyclic siloxanes and their oxidation products

**DOI:** 10.5194/acp-17-8357-2017

**Published:** 2017-07-10

**Authors:** Nathan J. Janechek, Kaj M. Hansen, Charles O. Stanier

**Affiliations:** 1 Department of Chemical and Biochemical Engineering, University of Iowa, Iowa City, IA 52242, USA; 2 IIHR Hydroscience and Engineering, University of Iowa, Iowa City, IA 52242, USA; 3 Department of Environmental Science, Aarhus University, Roskilde, Denmark

## Abstract

Cyclic volatile methyl siloxanes (cVMSs) are important components in personal care products that transport and react in the atmosphere. Octamethylcyclotetrasiloxane (D_4_), decamethylcyclopentasiloxane (D_5_), dodecamethylcyclohexasiloxane (D_6_), and their gas-phase oxidation products have been incorporated into the Community Multiscale Air Quality (CMAQ) model. Gas-phase oxidation products, as the precursor to secondary organic aerosol from this compound class, were included to quantify the maximum potential for aerosol formation from gas-phase reactions with OH. Four 1-month periods were modeled to quantify typical concentrations, seasonal variability, spatial patterns, and vertical profiles. Typical model concentrations showed parent compounds were highly dependent on population density as cities had monthly averaged peak D_5_ concentrations up to 432ngm^−3^. Peak oxidized D_5_ concentrations were significantly less, up to 9ngm^−3^, and were located downwind of major urban areas. Model results were compared to available measurements and previous simulation results. Seasonal variation was analyzed and differences in seasonal influences were observed between urban and rural locations. Parent compound concentrations in urban and peri-urban locations were sensitive to transport factors, while parent compounds in rural areas and oxidized product concentrations were influenced by large-scale seasonal variability in OH.

## 1 Introduction

Cyclic volatile methyl siloxanes (cVMSs) are present in a wide range of personal care and cosmetic products (e.g., hair products, lotions, antiperspirants, makeup, and sunscreens) as well as in sealers, cleaning products, and silicone products (Wang et al., 2009; Horii and Kannan, 2008; Dudzina et al., 2014; Lu et al., 2011; Capela et al., 2016). As high production volume chemicals (>1000tyr^−1^ produced) (OECD Environment Directorate, 2004), their environmental fate is an important topic. The most prevalent cVMS species in personal care products is decamethylcyclopentasiloxane (D_5_), although octamethylcyclotetrasiloxane (D_4_) and dodecamethylcyclohexasiloxane (D_6_) are also emitted (Horii and Kannan, 2008; Dudzina et al., 2014; Wang et al., 2009; Lu et al., 2011). Atmospheric lifetimes (Atkinson, 1991) are approximately 5–10 days at typical hydroxyl radical (OH) concentrations; accordingly, long-range transport (Xu and Wania, 2013; Krogseth et al., 2013a; McLachlan et al., 2010; Genualdi et al., 2011; MacLeod et al., 2011) of cVMS occurs. The environmental fate and transport of cVMS has been widely studied due to concerns of potential persistent, bioaccumulative, and toxic (PBT) behavior in the environment; however, assessing the environmental risk has been a subject of debate due to unique cVMS properties, evolving scientific information on properties and presence in the environment, and different interpretations of risk assessment information. The parent cyclic siloxanes have been the subject of a number of regulatory screenings including those by Canada (Environment Canada and Health Canada, 2008a, b, c), the UK (Brooke et al., 2009a, b, c), and the EU (ECHA, 2015); comprehensive review articles (Rucker and Kummerer, 2015; Wang et al., 2013) and recent environmental fate studies (Mackay et al., 2015; Gobas et al., 2015a, b; Fairbrother et al., 2015) are also relevant. The conceptual model of cVMS fate and transport is summarized as emission (mainly to the atmosphere) in population centers as a result of personal care product use (Mackay et al., 2015; Montemayor et al., 2013; Gouin et al., 2013), followed by atmospheric transport and reaction by OH (Xu and Wania, 2013). Emissions and concentrations are highly dependent on population, with urban locations (Yucuis et al., 2013; Genualdi et al., 2011; Krogseth et al., 2013b; Buser et al., 2013a; CompanioniDamas et al., 2014; Ahrens et al., 2014) and indoor environments (Tang et al., 2015; Yucuis et al., 2013; CompanioniDamas et al., 2014; Pieri et al., 2013; Tri Manh and Kannan, 2015) having much higher concentrations than remote locations. As this work shows, the population-dependent personal care product emissions are best validated for D_5_, and the importance of other emission types, as well as the variation in this by cVMS compound, is uncertain.

Substantial insights regarding cVMS fate, transport, and expected concentrations have come from atmospheric modeling studies. McLachlan et al. (2010) simulated atmospheric D_5_ concentrations using the Danish Eulerian Hemispheric Model (DEHM), a hemispheric-scale 3-D atmospheric chemistry and transport model. MacLeod et al. (2011) simulated D_5_ globally using the BErkeley-TRent Global Model (BETR Global), a multimedia mass balance model at 15^◦^ horizontal resolution. Global zonally averaged modeling using the multimedia GloboPop model has also been performed (Xu and Wania, 2013; Wania, 2003). Emission estimates have been back-calculated from measured atmospheric concentrations using a multimedia model (Buser et al., 2013a, 2014), and compartmental model studies focusing on specific partitioning or loss processes have also been conducted (Navea et al., 2011; Whelan et al., 2004). These modeling studies have permitted extension, both in time and space, beyond the sparse measurement dataset and testing of key model processes (emissions, fate, and transport) versus modeled concentrations. Latitudinal gradients, urban–rural– remote gradients, seasonal patterns, sensitivity to processes and parameterizations, and diel cycles have been explored using these models. Modeling studies have shown the largescale concentration patterns with OH as a dominant loss process, and quantified the importance of the atmosphere (relative to sediment and surface waters) for fate and transport. Seasonal and latitudinal trends can be explained in part by availability of OH. Models estimate D_5_ concentrations of 50ngm^−3^ and higher in well-mixed urban air (Navea et al., 2011), while 0.04–9ngm^−3^ is reported from models for remote locations (Krogseth et al., 2013a).

Atmospheric measurements of cyclic siloxanes have been performed in ambient air (McLachlan et al., 2010; Genualdi et al., 2011; Yucuis et al., 2013; Ahrens et al., 2014; Kierkegaard and McLachlan, 2013; Krogseth et al., 2013b, a; Buser et al., 2013a; Companioni-Damas et al., 2014). Higher concentration microenvironments have also been surveyed through measurement (wastewater treatment plants, landfills, and indoor air) (Krogseth et al., 2013b; Cheng et al., 2011; Wang et al., 2001; Pieri et al., 2013; Yucuis et al., 2013; Tri Manh and Kannan, 2015; Companioni-Damas et al., 2014; Tang et al., 2015). In several instances, model–measurement comparison has been conducted and, to a large extent, confirmed our understanding of emissions, fate and transport. Generally good agreement for rural and remote locations have been observed (McLachlan et al., 2010; Krogseth et al., 2013a; MacLeod et al., 2011; Navea et al., 2011; Xu and Wania, 2013; Genualdi et al., 2011), while urban areas tend to be underpredicted (Genualdi et al., 2011; Yucuis et al., 2013; Navea et al., 2011). Measured seasonal concentration variations have been replicated for sites in rural Sweden and the remote Arctic. However, it was noted that the DEHM tended to have better agreement during late spring (McLachlan et al., 2010) and late summer (Krogseth et al., 2013a) compared to winter. The BETR model conversely had better agreement during winter compared to late spring for the same rural Sweden site (MacLeod et al., 2011).

The majority of modeling and chamber study investigations, and all of the ambient measurements for cVMS, have focused on the emitted or “parent” cVMS compounds (i.e., D_4_, D_5_, and D_6_). The identity and fate of the cVMS oxidation products has received less scrutiny until recently, compared to the parent compounds. Sommerlade et al. (1993) reacted D_4_ with OH in an environmental chamber and identified multiple reaction products by GC-MS, with the single OH substituted silanol (D_3_TOH) as the most prevalent resolved species, with species identification confirmed by matching retention time and mass spectra compared to synthesized D_3_TOH. Because of the method of collection (the product was collected from rinsing the environmental chamber walls with solvent) confirmation of secondary *aerosol production* from D_4_ oxidation was not possible from Sommerlade et al. (1993). Chandramouli and Kamens (2001) reacted D_5_ in a smog chamber, with separate analysis of gas and aerosol products, confirming the presence of D_4_TOH in the GS/MS analysis of the condensed aerosol phase.

Wu and Johnston (2016) conducted more exhaustive characterization of aerosols from photooxidation of D_5_, using high-performance mass spectrometry, revealing both monomeric and dimeric oxidation products, with molar masses up to 870. Oxidation progressed not only by substitution of a methyl group with OH (e.g., leading to D_4_TOH) but also by substitution with CH_2_OH; linkages between SiO rings to form dimers were through O, CH_2_, and CH_2_CH_2_ linkage groups.

Aerosols containing Si and likely from photooxidation of gaseous precursors have been recently identified in multiple locations in the US using laser ablation particle mass spectrometry of ultrafine particles (Bzdek et al., 2014). Bzdek et al. (2014) contend that a photooxidation source is most consistent with observations because of the times of day of occurrence, short atmospheric lifetime of the particle size in question (10–30nm), lack of wind direction dependence that would be expected from primary sources, ubiquity across disparate measurement sites, and similarity in temporal evolution of nanoaerosol Si to other species with known photochemical sources. Except for the reports of the concentrations of ambient oxidized cVMS in Bzdek et al. (2014), there are no ambient measurements or model-based estimates of the potential aerosol concentrations from cVMS oxidation. This work begins to address that gap by simulating the gas-phase oxidation product concentrations using the atmospheric chemistry and transport model Community Multiscale Air Quality (CMAQ). As experimental determinations of aerosol yield become available, the simulations can be updated to include secondary organosilicon aerosol concentrations.

This work builds on the limited information available on the oxidation products. Properties relevant to fate and transport (e.g., Henry’s law coefficient) have been predicted in this work and in others based on structure activity relationships (Buser et al., 2013b; Whelan et al., 2004). Latimer et al. (1998) measured equilibrium gas–particle partitioning of D_5_ and D_4_TOH on diesel, wood, coal soot, and Arizona fine dust aerosols. Whelan et al. (2004) performed equilibrium air–particle and air–cloud droplet partitioning modeling of multiple substituted OH silanols. More extensive information is available about the gas–particle partitioning (Latimer et al., 1998; Tri Manh and Kannan, 2015; Tri Manh et al., 2015; Kim and Xu, 2016) and aerosol-phase reactions (Navea et al., 2011, 2009a, b) of the precursor compounds, but these confirm that the gas-phase oxidation and transport of the parent compounds are substantially more important than the heterogeneous oxidation pathways and thermodynamic partitioning of the parent compounds onto ambient aerosols.

In this work, atmospheric gas-phase concentrations of D_4_, D_5_, D_6_, and its oxidization products are modeled comprehensively using the CMAQ chemical transport model. The purpose of the model-based investigation is twofold. First, it enables the highest resolution (36km) simulation to date of the parent compound over the US; the model simulates vertical profiles, urban-to-rural transitions, and the dependence of these on factors such as season and mixed layer height. Second, this paper reports, for the first time in detail, concentrations of the cVMS oxidation products. Some fraction of products is likely distributed into the aerosol phase, thus contributing to aerosol Si concentrations on regional and global scales. We expand upon the modeling first presented in Bzdek et al. (2014), but with improved emission estimates, inclusion of wet and dry deposition, and incorporation of season-dependent boundary conditions.

## 2 Methods

Cyclic siloxanes and oxidized cyclic siloxanes were modeled with the 3-D atmospheric chemical transport model CMAQ (Byun and Schere, 2006), modified to include cyclic siloxane species. CMAQ version 4.7.1 was used and the modeling domain covered the contiguous US, northern Mexico, and southern Canada. The domain had 14 vertical layers and a horizontal resolution of 36km. Four 1-month simulations were performed for January, April, July, and October to characterize seasonal variability in cyclic siloxane atmospheric concentrations. A spin-up period of 7 days was used to minimize the influence of zero initial conditions for the cyclic siloxanes species. Meteorology was from the Weather Research and Forecasting (WRF) model version 3.1.1 for the meteorological year of 2004. WRF was run with time steps of 120s, 30 vertical layers, the Morrison double-moment microphysics scheme, the RRTMG longwave and shortwave physics scheme, the Pleim–Xiu surface layer, the Pleim–Xiu land surface model with two soil layers, and the ACM2 planetary boundary layer (PBL) scheme. Reanalysis nudging using North American Regional Reanalysis (NARR) data was performed every 3h.

The cyclic siloxanes were added to the CMAQ model by adding D_4_, D_5_, D_6_, and the oxidized species, o-D_4_, oD_5_, and o-D_6_ to the cb05cl_ae5_aq mechanism. Rate constants for the parent cyclic siloxanes reacting with OH were used from Atkinson (1991), where D_4_ and D_5_ were determined experimentally and D_6_ estimated from the reported D_5_ per methyl rate. The rate constants used were 1.01×10^−12^, 1.55×10^−12^, and 1.92×10^−12^ cm^3^ molecule^−1^ s^−1^ for D_4_, D_5_, and D_6_, respectively. Reactions of the oxidation products are not included in the model. In part, this is because information is limited on the kinetics of further oxidation and on the changes that this would cause for fate, transport, and properties. Whelan et al. (2004) modeled subsequent oxidation reactions, and chamber-based oxidation studies observe multiple substitution products likely due to multiple substitution reactions or auto-oxidation by internal rearrangement (Wu and Johnston, 2016). In the model, only the first oxidation is computed. The oxidation products are denoted o-D_4_, o-D_5_, and o-D_6_, and for calculation of physical properties relevant to deposition, the single OH substitution is assumed.

Wet and dry deposition of the primary species (e.g., D_4_, D_5_) were added to the model using Henry’s law coefficients (Xu and Kropscott, 2012). For the oxidized cyclic siloxanes, physicochemical parameters were estimated using EPI Suite HENRYWIN v3.20 (EPA, 2012) for the single OH substitution of one methyl group of the parent cyclic siloxane (e.g., D_3_TOH, D_4_TOH). Deposition-related inputs necessary for the CMAQ deposition routine included Henry’s law coefficients, mass diffusivities, reactivity, and mesophyll resistance. CMAQ calculates dry deposition as a deposition velocity (dependent on mixing/turbulence, molecular properties, and land type) multiplied by the lowest model layer concentration (Byun et al., 1999), and wet deposition using Henry’s law coefficients and precipitation rates (Roselle and Binkowski, 1999). Dry deposition therefore treats the surface as an infinite sink, which is consistent with other species in the model. The mass diffusivity values were calculated by the Fuller, Schettler, and Giddings (FSG) method (Lyman et al., 1982), where molar volume was estimated based on element contributions. Sulfur molar volume contribution values were substituted for silicon atoms since silicon values were not available. Calculated mass diffusivity values, as estimated by the FSG method were 0.0512 (D_4_), 0.0454 (D_5_), 0.0411 (D_6_), 0.0527 (o-D_4_), 0.0464 (o-D_5_), and 0.0419cm^2^ s^−1^ (o-D_6_). The reactivity parameter was set at 2.0 in common with methanol and other species of limited reactivity. The mesophyll resistance, which is used to account for uptake by plants, was set to zero (only a few species had mesophyll resistances specified in CMAQ, such as NO_2_, NO, CO, and Hg gas). Molecular weight for the oxidized cyclic siloxanes assumed the single substituted OH species. The molecular weight of D_6_ and o-D_6_ exceeded the limit of the CMAQ dry deposition routine m3dry (390gmol^−1^) and values in excess of the limit were set to the limit. The impact of this substitution is expected to be minimal, since it is a minor adjustment to a minor pathway; dry deposition of cVMS is relatively small (McLachlan et al., 2010; Xu and Wania, 2013; Whelan et al., 2004).

Emissions of cyclic siloxanes were distributed according to gridded population for the US, Canada, and Mexico, while Caribbean countries were neglected. The US, Canadian, and Mexican per capita emission rates of D_5_ provided by personal communication (R. van Egmond, personal communication, 2013) and previously used and reported in McLachlan et al. (2010) were adopted for this study. Briefly, as reported in McLachlan et al. (2010), D_5_ emission rates were derived from country-specific market share based on antiperspirant sales data combined with D_5_ consumption data from antiperspirant plus 10% to account for other sources. A table of many available cVMS emissions rates from multiple methods are represented in Table S2, and a wide variation exists. To calculate D_4_ and D_6_ emission rates, ambient measurements from Chicago (Yucuis et al., 2013) were used to estimate emission ratios relative to D_5_. Chicago was chosen since it is a major urban area and atmospheric measurements should be most fresh and therefore the best representation of emission rates. However, since OH reactivity (and other fate and transport properties) vary from compound to compound, ambient measurements of compound ratios will not match emission ratios, except in air parcels that are so fresh as to have seen no oxidation. To check for the influence of air mass aging in the measurements of Yucuis et al. (2013), the ratio NO_*x*_ /NO_*y*_ was used as a marker of air mass age (Slowik et al., 2011). This ratio is high in fresh emissions, and decreases as the air mass is oxidized. Hourly measurements of NO_*x*_ and NO_*y*_ from Northbrook, Illinois (EPA), were inspected during the time period of the Chicago sampling in Yucuis et al. (2013). Using the NO_*x*_ /NO_*y*_ photochemical age estimate, we calculated that emitted ratios vs. ambient ratios likely differed by less than 1% (see Supplement). The Chicago cyclic siloxane measurements were therefore used as emission ratios without photochemical age correction. The resulting emission ratios, 0.243 and 0.0451 for D_4_ /D_5_ and D_6_ /D_5_, respectively, were multiplied by the D_5_ emission rate to estimate the D_4_ and D_6_ emission rates. The resulting D_4_, D_5_, and D_6_ country emission rates, which were constant for all simulations, were multiplied by gridded population and merged with year 2004 emissions generated by the Sparse Matrix Operator Kernel Emissions (SMOKE) model version 2.5. Population data were from census-derived population surrogates from EPA 2011 v6.0 Air Emissions Modeling Platform and are based on permanent residency and does not include seasonal tourism. This may cause inaccuracies in emissions near parks and other tourist destinations. SMOKE emissions were calculated from NEI 2002, version 3, with on-road and point sources projected to 2004 using EGAS, the EPA’s point source and economic growth analysis system. Biogenic emissions were from BEIS 3.13.

Boundary conditions were from previous DEHM modeling that modeled D_5_ concentrations using 2009 emission rates as described above (Hansen et al., 2008; McLachlan et al., 2010). The DEHM was run for the Northern Hemisphere at 150km resolution. We extracted the D_5_ concentrations from the DEHM for year 2011 meteorology along our model boundary. Boundary concentrations were horizontally and vertically resolved, varied by month, but were time invariant within each month. Since the DEHM only included D_5_, D_4_ and D_6_ concentrations were estimated using measurement ratios taken from a background site at Point Reyes, California (Genualdi et al., 2011). Point Reyes samples had ratios of 0.646 and 0.0877 for D_4_ /D_5_ and D_6_ /D_5_, respectively. The background ratios combined with the “fresh” emission ratios (described previously) were used to calculate a photochemical age. The calculation of a photochemical age was necessary since the siloxanes have different OH reaction rates and therefore the siloxane ratios change with season due to varying OH concentrations. Using this method, we calculated an age of 17.6 days using the D_4_ /D_5_ ratios, and this is the age used for further calculations. The calculated photochemical age was combined with season-specific OH concentrations (Spivakovsky et al., 2000) to calculate monthly resolved D_4_ /D_5_ and D_6_ /D_5_ “background” ratios. These monthly resolved D_4_ /D_5_ and D_6_ /D_5_ ratios were then used for the entire model boundary. Additional details are available in the Supplement.

## 3 Results and discussion

### 3.1 Spatial variation in concentrations

**Figure 1.**
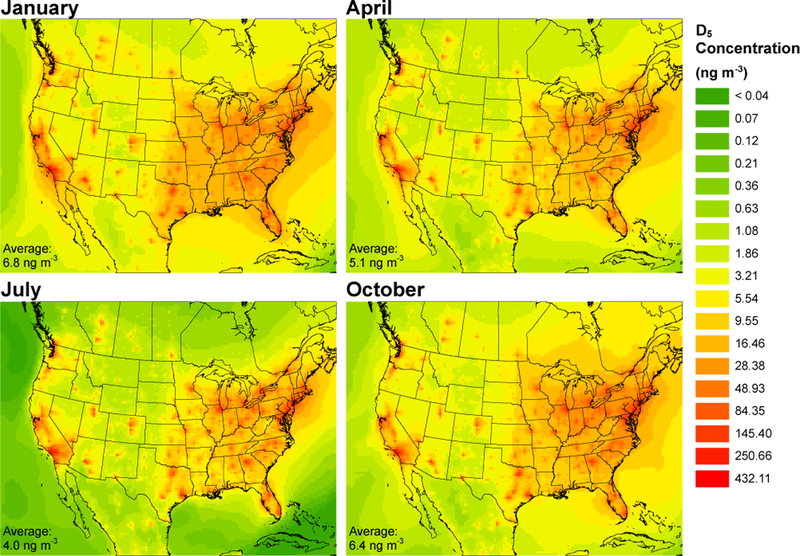
Monthly averaged surface layer D_5_ concentrations. The domain average concentration is shown in the lower left for each month.

**Figure 2.**
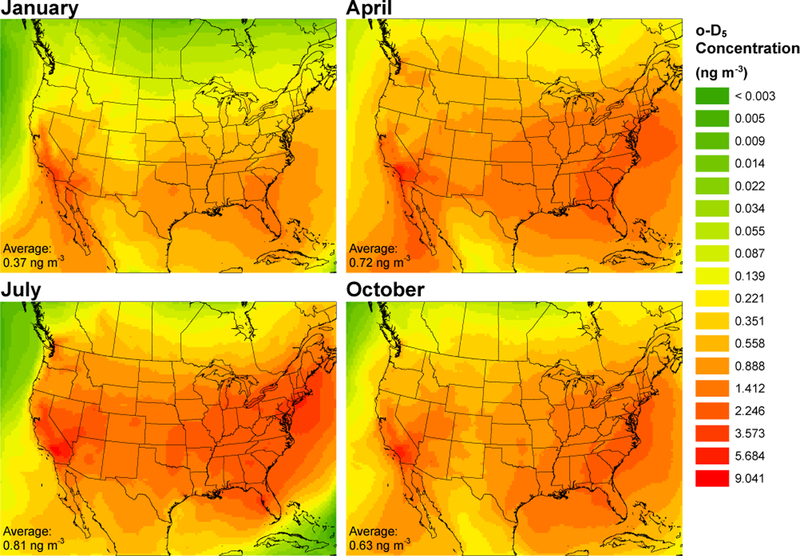
Monthly average surface layer oxidized D_5_ (o-D_5_) concentrations. The domain average concentration is shown in the lower left for each month.

Figures 1 and 2 show the 30-day averaged D_5_ and oxidized D_5_ (o-D_5_) modeled concentrations for January, April, July, and October. The spatial distribution of cVMS and oxidized cVMS compounds show a strong population dependence with major urban areas having elevated D_5_ concentrations and peak o-D_5_ concentrations occurring hundreds of km downwind of source regions due to the time it takes for the parent compounds to react with OH. Table 1 displays the monthly minimum, maximum, and average concentrations for the entire modeled domain. The 36km grid cell with the highest 30-day average surface concentration of D_5_ was 432, 379, 301, and 265ngm^−3^ for January (Los Angeles – Long Beach), April (Los Angeles – Long Beach), October (New York City), and July (New York City), respectively. The domain-averaged surface concentrations of D_5_ were 6.82, 6.43, 5.09, and 4.04ngm^−3^ for January, October, April, and July. Simulated o-D_5_ was much lower than simulated D_5_ concentrations. For example, the 36km grid cell with the highest 30-day average surface concentration of o-D_5_ was 9.04, 5.21, 4.86, and 3.19ngm^−3^ for July (NE of Los Angeles – Victorville), October (E of Los Angeles – San Bernardino), April (SE of Los Angeles – Mission Viejo), and January (Los Angeles – Long Beach), respectively. The domain average surface concentration for o-D_5_ was 0.81, 0.72, 0.63, and 0.37ngm^−3^ for July, April, October, and January, respectively. The peak domain-averaged concentrations occurred during January for D_5_ and July for o-D_5_, which is expected based on seasonal trends of OH in North America (Spivakovsky et al., 2000).

**Figure 3.**
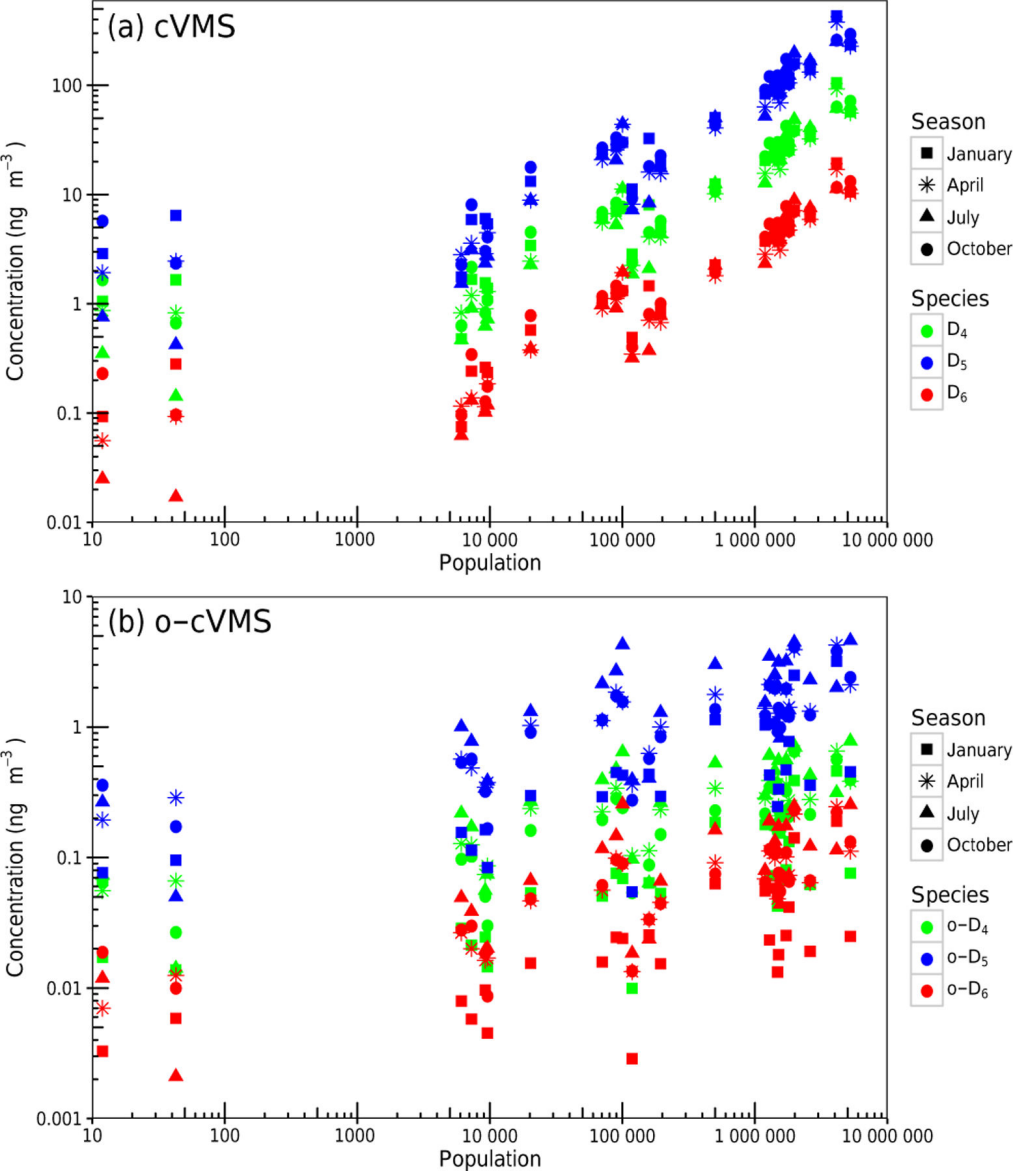
Average monthly CMAQ modeled surface (**a**) cVMS and (**b**) oxidized cVMS concentrations are plotted versus 36km grid cell population for 26 US and Canadian sites. These sites include the 10 most populous US metropolitan areas, previous siloxane measurement sites, and NOAA Climate Monitoring and Diagnostics Laboratory (CMDL) sites. See Table S3 for the listing of these sites.

Figure 3 shows the monthly averaged cVMS and oxidized cVMS concentrations versus the model grid cell population for 26 US and Canadian sites. These sites include the most populous 10 US metropolitan areas, siloxane measurement sites, and NOAA Climate Monitoring and Diagnostics Laboratory (CMDL) sites; see Table S3 for the full list. Modeled concentrations are strongly dependent on population, with New York City and Los Angeles having the highest concentrations (Table S4). In addition to the population dependence, concentrations were greatest for D_5_ followed by D_4_ and D_6_. This follows from our assumed emission ratios and agrees with North American measurement data (Yucuis et al., 2013; Genualdi et al., 2011; Ahrens et al., 2014; Krogseth et al., 2013b). The prevalence of D_4_ relative to D_6_ is of interest because analysis of cVMS composition in consumer products (Horii and Kannan, 2008; Wang et al., 2009; Dudzina et al., 2014; Lu et al., 2011; Capela et al., 2016) suggests that D_6_ is more abundant than D_4_ – while in our modeling (and atmospheric measurements) D_4_ concentrations are higher than D_6_ concentrations. Four explanations bear further investigation: (1) non-personal-care emissions (e.g., cVMS residuals from polymer production) may play a more important role for D_4_ than other species based on UK emission estimates (Brooke et al., 2009a, b, c), (2) possible siloxane conversion during sample collection (Kierkegaard and McLachlan, 2013; Krogseth et al., 2013a), (3) higher D_4_ volatility (Lei et al., 2010) could cause both more difficult detection in personal care products and a larger fraction volatilization from products, and (4) uncertainty and/or spatiotemporal variability in the D_4_ /D_5_ and D_6_ /D_5_ ratios from ambient measurements in Chicago used to extend the D_5_ emissions estimates to D_4_ and D_6_.

#### 3.1.1 Seasonal variation in concentrations

Since OH concentrations vary seasonally we expect higher cVMS in the winter (low OH) and lower in the summer (high OH). This has been supported by previous measurement studies. For example, McLachlan et al. (2010) measured D_5_ at a rural site in Sweden (59^◦^ N) and observed reduced D_5_ concentrations for the period of May–June compared to January–April. Measurements in a remote Arctic location (79^◦^ N) observed higher concentrations in the winter compared to late summer (Krogseth et al., 2013a). For OH concentrations to influence cVMS concentrations, time for oxidation is required – so the relationship between seasonal OH and cVMS is expected at receptor sites where most cVMS is transported from upwind locations. At sourcedominated locations, the influence of OH should be limited. For example, studies from Toronto highlight local meteorological influences as important in determining variation in siloxane (D_3_–D_6_) concentrations (Ahrens et al., 2014; Krogseth et al., 2013b).

Figure 1 shows similar D_5_ spatial distribution between the 4 months, especially for urban areas. Domain peak and average concentrations (Table 1) have highest concentrations in January and lowest in July which agree with seasonal OH concentrations, but specific grid cells (particularly urban locations) often deviate from this. Rural and remote locations are more likely to follow the OH-induced seasonal pattern. Seasonal variation for the 26 sites in Table S3 was examined using patterns in the month of highest concentration. Sites were classified as either urban or rural based on summer D_5_ concentrations. For urban sites, the most prevalent month with highest average D_5_ concentration was October (59%), followed by July (23%) and January (18%). Restricting the analysis to the rural sites (summer D_5_ concentration below 17ngm^−3^), peak D_5_ concentrations occurred in January (56%), followed by October (33%) and April (11%). The month of lowest average D_5_ concentrations occurred in July for 100% of the rural sites and 24% of the urban sites. Similarly, looking at the breakdown for the monthly averaged oxidized D_5_ concentrations, highest concentrations generally occurred in July, which was true for 73% of the 26 sites. Figure 2 shows differences in the spatial distribution of o-D_5_ between months. The analyzed sites therefore suggest less of a seasonal trend for the parent compounds as compared to the oxidized products, and there are differences in seasonal trends between source and non-source locations. Remote and rural sites are more dependent on lifetime with respect to reaction with OH, while source locations are less sensitive. This agrees with previous modeling which showed reduced seasonal variability in D_5_ concentrations for urban areas compared to remote locations (McLachlan et al., 2010; MacLeod et al., 2011; Xu and Wania, 2013).

Statistical relationships between D_5_, OH, PBL height, and wind speed (WS) were explored using least squares multiple linear regression. For the 26 analyzed sites, OH, PBL, and WS values were normalized to their summer values and then used as predictive variables of the ratio of D_5_ in each season to its summer value at the same site. In other words, the regression analysis is testing the local seasonto-season variability across seasons and sites (e.g., whether winter:summer D_5_ concentration is correlated with winter:summer OH^−1^). Sites were split between urban and rural as described previously. For urban sites, D_5_ concentration was only correlated to OH^−1^ when WS^−1^ was also included, with WS being the dominant variable. The strongest predictive variables were PBL^−1^ and WS^−1^ with an adjusted *R*^2^ fit of 0.50 and a *p* value of <0.001. The regression analysis supports the previous conclusion: ventilation of local emissions through PBL height and local winds is the strongest influence on urban siloxane concentrations.

For the rural sites, WS^−1^ was the only variable of significance but had a low adjusted *R*^2^ of 0.10, *p* value of 0.056, and a negative coefficient meaning lower wind speed results in lower D_5_ concentrations. Repeating the linear regression, excluding Canadian sites and Point Reyes (California), led to similar results. Canadian sites were excluded since nonsiloxane Canadian emissions were allocated by population and may cause errors in OH due to misallocation of nitrogen oxides and reactive organic gases from some source sectors (Spak et al., 2012). Point Reyes was excluded due to high grid cell population despite low D_5_ concentrations. See Supplement for additional regression results. From this analysis, we conclude that factors other than local OH and local meteorology control rural/remote siloxane concentrations. These factors likely include regional OH and regional transport patterns.

### 3.2 Model–measurement comparison

The model results were compared to measurement values in the Midwest (Yucuis et al., 2013), North American measurements from the Global Atmospheric Passive Sampling (GAPS) network (Genualdi et al., 2011), and several Toronto measurements (Genualdi et al., 2011; Ahrens et al., 2014; Krogseth et al., 2013b).

#### 3.2.1 Midwest model comparison

In Yucuis et al. (2013) measurements were taken at three Midwest locations during the summer (June–August) of 2011. The measurements were collected, in duplicate, at sites with varying population density. Measurements from Chicago, Illinois, were collected consecutively as sixteen 12h samples from 13 to 21 August; from Cedar Rapids, Iowa, as four 24h samples non-consecutively from 29 June to 26 July; and from West Branch, Iowa, as five samples that ranged from 30 to 47h on 6 July and consecutively from 15 to 22 July. The measurements were compared to the 1– 30 July modeled hourly concentrations averaged as 12, 24, and 36h intervals for the Chicago, Cedar Rapids, and West Branch sites, respectively. These sampling periods and sample counts are insufficient to establish representativeness of the values as monthly or seasonal averages. The model results were averaged using time of day and duration matching the measurements but do not correspond to the exact measurement days or meteorology. Measurements are from 2011 and the model’s meteorological fields are from 2004; however, average wind speeds, wind directions, and boundary layer heights are typically similar from year to year.

**Figure 4.**
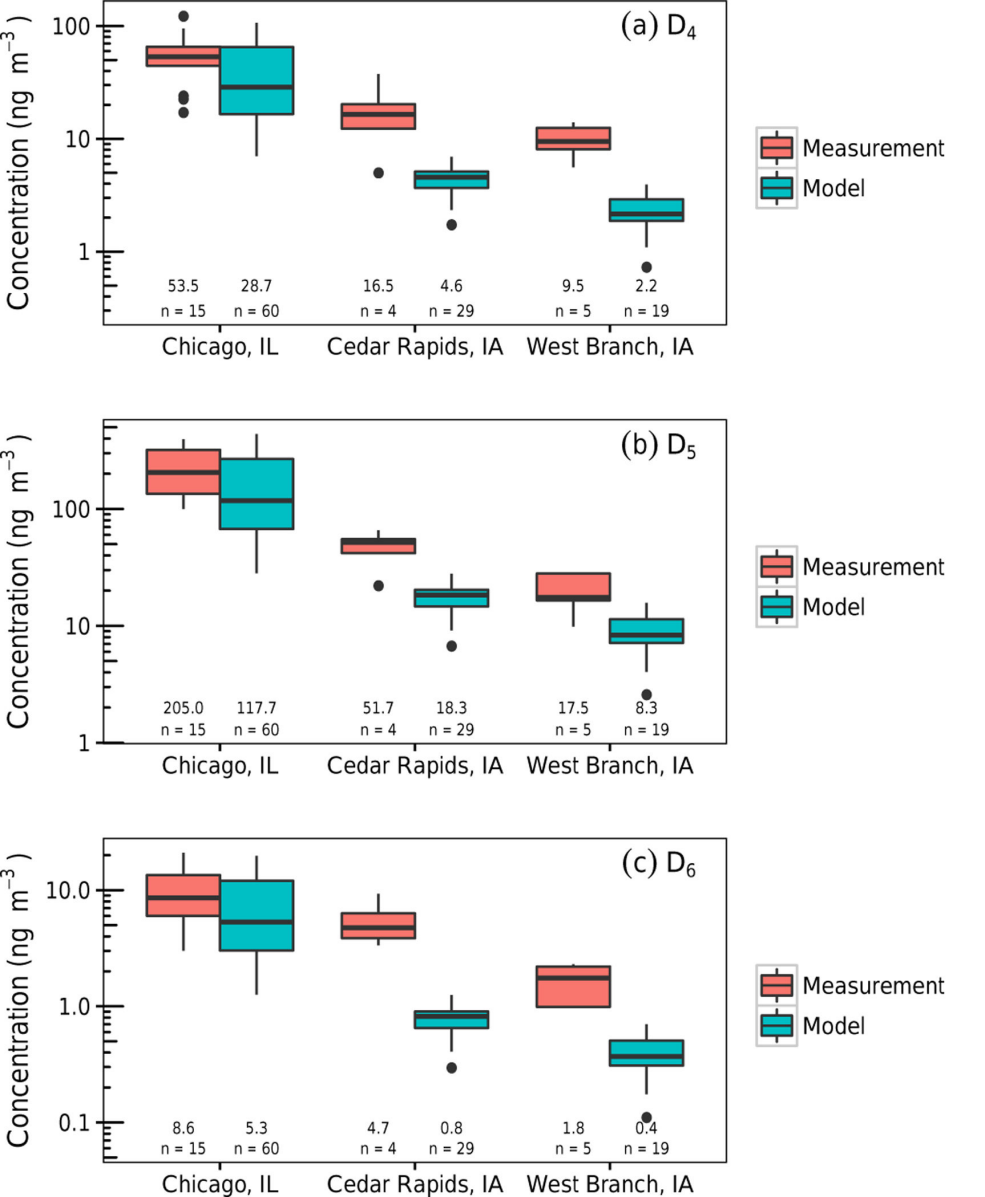
Model comparison to Yucuis et al. (2013). Model results from CMAQ (1–30 July simulation); measurements were conducted in 2011 from 13 to 21 August (Chicago), 29 June to 26 July (Cedar Rapids), and 6 to 22 July (West Branch), respectively. Hourly model data were averaged to 12, 24, and 36h periods, starting at typical measurement start times. Median concentrations and number of observations are tabulated under the box plots.

Figure 4 displays the box plot comparison of the three Midwest sites of Yucuis et al. (2013) and the modeled concentrations. The model does capture the population dependence that the measurements show, with Chicago observing highest concentrations followed by Cedar Rapids and West Branch. Modeled concentrations, however, are lower for all three locations compared to the measurements with fractional bias (Table S10) at Chicago of −0.31, −0.31, and −0.28 (for D_4_, D_5_, and D_6_, respectively); Cedar Rapids of −1.25, −0.93, and −1.51; and West Branch of −1.25, −0.78, and −1.23. Comparing the relative percent error of the mean modeled concentrations to the measured values, we found that Chicago sites had relative percent errors of around 25%, while the other sites had values ranging from 56 to 86%. For Chicago, error between the species was similar and this is most likely the result that D_4_ and D_6_ emission rates were calculated based on the Chicago measurements. For Cedar Rapids and West Branch, D_5_ had the lowest error, while D_4_ and D_6_ were larger. This may indicate that the siloxane emission ratios vary based on location.

One possible explanation for low model concentrations could be low emission estimates. Current emission estimates (Table S2) vary considerably and the estimates used in this work were 32.8, 135, and 6.10mgperson^−1^ day^−1^ for D_4_, D_5_, and D_6_, respectively, for the US and Canada, while the Mexico emissions were 5.92, 24.4, and 1.10mgperson^−1^ day^−1^ for D_4_, D_5_, and D_6_. Previous emission estimates have ranged 0.001–100, 0.002–1200, and 0.0009–80mgperson^−1^ day^−1^ for D_4_, D_5_, and D_6_, respectively (Tang et al., 2015; Buser et al., 2013a, 2014; Navea et al., 2011; Yucuis et al., 2013; Horii and Kannan, 2008; Dudzina et al., 2014; Wang et al., 2009; Capela et al., 2016). Additionally, non-personal-care product emissions could be important, as could potential geographical, demographical, or temporal influences on siloxane emissions. As datasets of cVMS concentrations, particularly those with simultaneous values for D_4_, D_5_ and D_6_, become available in more sourceoriented locations and seasons, the emissions estimates, particularly for D_4_ and D_6_, should be refined.

The treatment of deposition as an infinite sink could also cause low gas-phase concentrations (deposition overpredicted) if surface concentration are not degraded quickly. Experimental studies show the parent cVMS degradation is slow in soil (Wang et al., 2013); however, this is likely minimized due to low deposition potential as predicted by high air–water (*K*_AW_) and low octanol–air (*K*_OA_) partitioning coefficients (Xu and Wania, 2013). Octanol–air (log*K*_OA_) partitioning values, which is an indication of the ability to partition to soil and plants (Shoeib and Harner, 2002), are 4.29– 5.86 for D_4_–D_6_ (Xu and Kropscott, 2012), which is similar to or higher than other organic species with modeled deposition such as methanol, aldehydes, and carboxylic acids. The oxidized species are likely more sensitive due to greater deposition potential as EPI Suite predicts lower log*K*_AW_ and higher log*K*_OA_ values, however the surface degradation kinetics of the oxidation products are not known.

#### 3.2.2 GAPS model comparison

**Figure 5.**
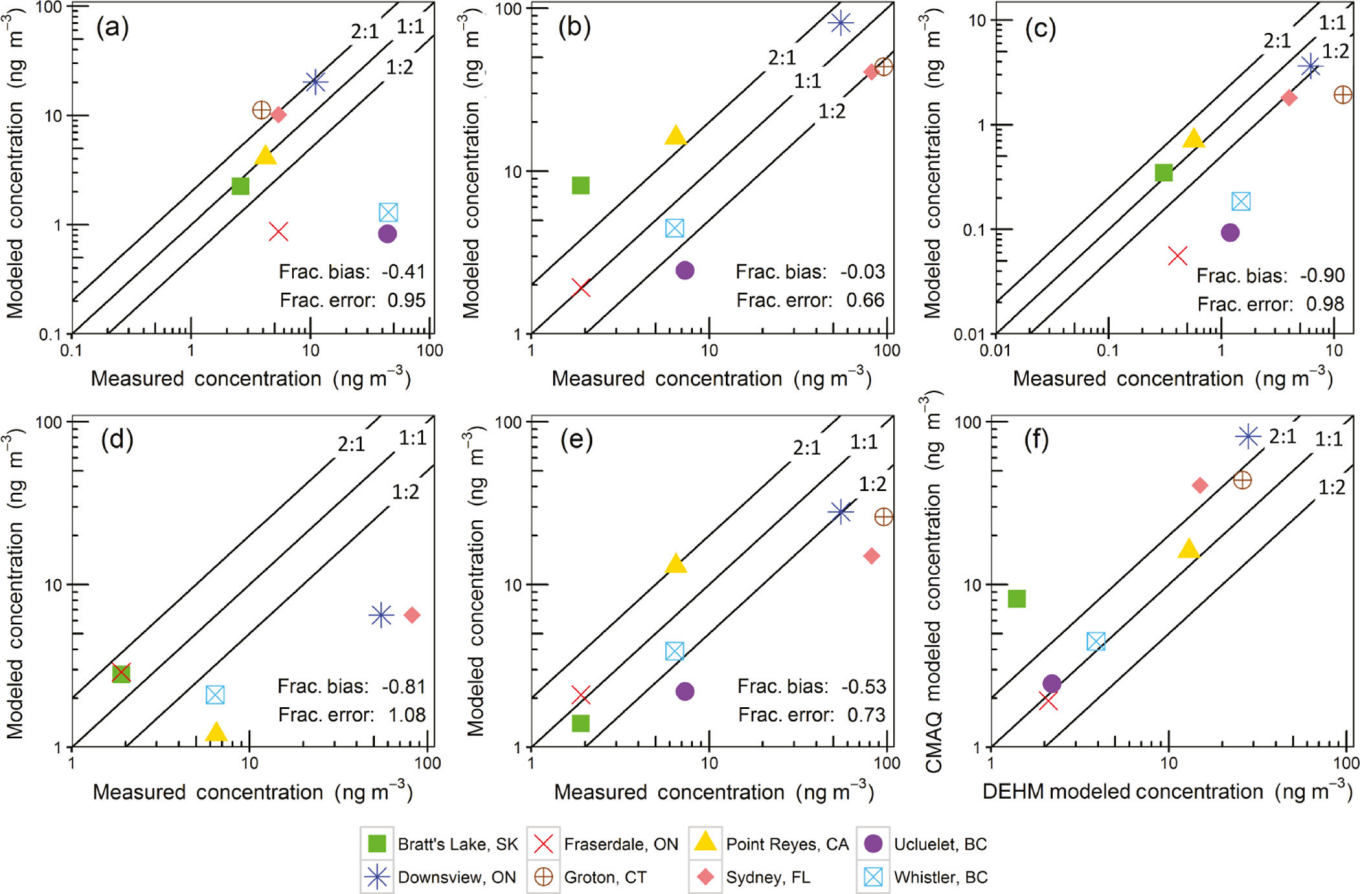
Plot (**a**) shows CMAQ D_4_, (**b**) CMAQ D_5_, (**c**) CMAQ D_6_, (**d**) BETR D_5_, and (**e**) DEHM D_5_ modeled concentrations compared to Genualdi et al. (2011) measurements. Plot (**f**) compares modeled CMAQ D5 versus DEHM D5 concentrations. CMAQ model results are the April averaged concentrations while BETR and DEHM model results are from Genualdi et al. (2011) and represent the same period as the measurements. Model resolution was 36km for CMAQ, 150km for DEHM, and 15^◦^ for BETR.

The model was also compared to measurements of Genualdi et al. (2011). These measurements were collected from passive samplers as part of the GAPS network over 3 months in 2009, generally from late March to early July. Figure 5 shows the CMAQ-modeled April versus measurements for eight locations within our domain. Again, as with the Yucuis et al. (2013) comparison, the modeled results do not explicitly represent meteorological conditions of the measurement period. Fractional error (Table S11) for D_4_ varied from 0.02 to 1.93, with Point Reyes having the lowest and Ucluelet the highest. For D_5_, fractional error values ranged from 0.02 to 1.24 with Fraserdale the lowest and Bratt’s Lake the highest. Similarly, for D_6_, the fractional error varied from 0.11 to 1.71 with Bratt’s Lake the lowest and Ucluelet the highest. Averaged over the eight sites, the overall fractional biases were −0.41, −0.03, and −0.90 for D_4_, D_5_, and D_6_, respectively. The mean fractional error was 0.95, 0.66, and 0.98 for D_4_, D_5_, and D_6_ species. Therefore, based on the fractional error values, D_5_ had the best agreement followed by D_4_ and D_6_. This is not surprising that D_5_ had the best agreement since D_4_ and D_6_ emission rates are estimated based on Chicago measurements and would have additional uncertainty compared to the D_5_ emission uncertainty.

On average, fractional bias for D_5_ was close to zero while D_4_ and D_6_ had greater negative bias due to significant deviations for Fraserdale, Ucluelet, and Whistler. Aside from these three sites, the D_4_ predictions generally agreed well with the measurements. These same three sites and Groton were also significantly underpredicted for D_6_, but other sites were within a factor of 2 of the measurements. Possible explanations for model deviation could be population errors (Ucluelet and Whistler are tourist destinations and the population dataset used did not include visitors), non-personal-care product emissions, or product transformation of higher-molecular-weight siloxanes to D_4_ on sampling media (Kierkegaard and McLachlan, 2013; Krogseth et al., 2013a), or that our boundary conditions could be underestimating Asian cVMS transport. Genualdi et al. (2011) hypothesized the high D_4_ concentrations measured at Whistler and Ucluelet could be due to transport from Asia since D_4_ concentrations were greatest at west coast locations and especially at high-altitude sites.

Model overprediction for D_5_ occurred for the Point Reyes and Bratt’s Lake sites. Representation error is a likely cause of this, since the actual sampling sites were upwind of large population centers (San Francisco and Regina, Saskatchewan) in these grid cells; at 36km resolution, the upwind sampling sites and the downwind emission centers are not resolved. However, Point Reyes and Bratt’s Lake D_4_ and D_6_ concentrations were close to the modeled values.

We also compare the 36km CMAQ D_5_ concentration results to values from the DEHM and BETR models. The BETR model did not report values for Ucluelet or Groton so those sites are not included. The D_5_ modeling attempts were ordered from most skilled to least skilled by using the mean of the fractional bias and fractional error (in parentheses) scores: CMAQ −0.03 (0.66), DEHM −0.53 (0.73), and BETR −0.81 (1.08). The CMAQ and DEHM models had similar performance for Fraserdale, Whistler, Ucluelet, and Point Reyes, while the urban areas (Downsview; Sydney, Florida; and Groton) were better predicted in the CMAQ model. Bratt’s Lake was overestimated compared to the DEHM and may have to do with the greater influence of Regina, Saskatchewan, emissions due to improved model resolution. The differences in modeled concentrations are most likely due to higher spatial resolution for CMAQ (36km) versus 150km (DEHM), and 15^◦^ (BETR) resolutions.

#### 3.2.3 Toronto model comparison

Multiple measurement and modeling studies have investigated cVMS concentrations in Toronto, Canada. Table 2 shows the mean and range of cVMS concentrations in Toronto for each of the 4 months as simulated by the CMAQ model. Table 2 further includes the March 2010–April 2011 measured concentrations as collected by both passive and active sampling (Ahrens et al., 2014), active sampling from March to June 2012 and passive sampling from July to October 2012 (Krogseth et al., 2013b), and passive sampling (April–June 2009) from the GAPS network (Genualdi et al., 2011). Finally, the BETR and DEHM modeled D_5_ concentrations (Apri–June 2009) are also tabulated (Genualdi et al., 2011). The CMAQ results compared favorably to the Ahrens et al. (2014) measurements, with CMAQ monthly averages that generally fell within the reported measurement concentration ranges. D_4_ monthly averages were within a factor of 0.97–1.94, D_5_ within a factor of 0.59–1.39, and D_6_ within a factor of 0.33–0.78 of the yearly averaged active and passive sampling measurements. Comparison of the range of concentrations showed that CMAQ 24h averaged ranges were 4.6–60.6 (D_4_), 17.1–247.7 (D_5_), and 0.74–11.13 (D_6_) ngm^−3^ compared to Ahrens et al. (2014) 24h active sampling range of 2.8–77 (D_4_), 15–247 (D_5_), and 1.9–22 (D_6_) ngm^−3^. Similarly, good agreement was observed for the active and passive sampling measurements from Krogseth et al. (2013b), average April CMAQ D_4_, D_5_, and D_6_ concentrations were a factor of 0.84, 0.88, and 0.67, respectively, of the measured average, the concentration ranges were similar, with higher peak concentrations occurring for the measurements despite sampling for 2–3 days. For the passive samples of Krogseth et al. (2013b), July and October average CMAQ concentrations were 0.69–0.76 for D_4_ and 0.95– 1.04 for D_5_ compared to the measurements. CMAQ April averages were 1.85, 1.49, and 0.59 times the Genualdi et al. (2011) measurements. Previous Toronto modeling predicted 6.5ngm^−3^ (BETR) and 28ngm^−3^ (DEHM), which were significantly lower than the spring CMAQ D_5_ concentration of 81.6ngm^−3^. Overall, the CMAQ model was able to better predict the higher observed concentrations of Toronto, which again can most likely be attributed to increased model resolution.

### 3.3 Compound ratios

**Figure 6.**
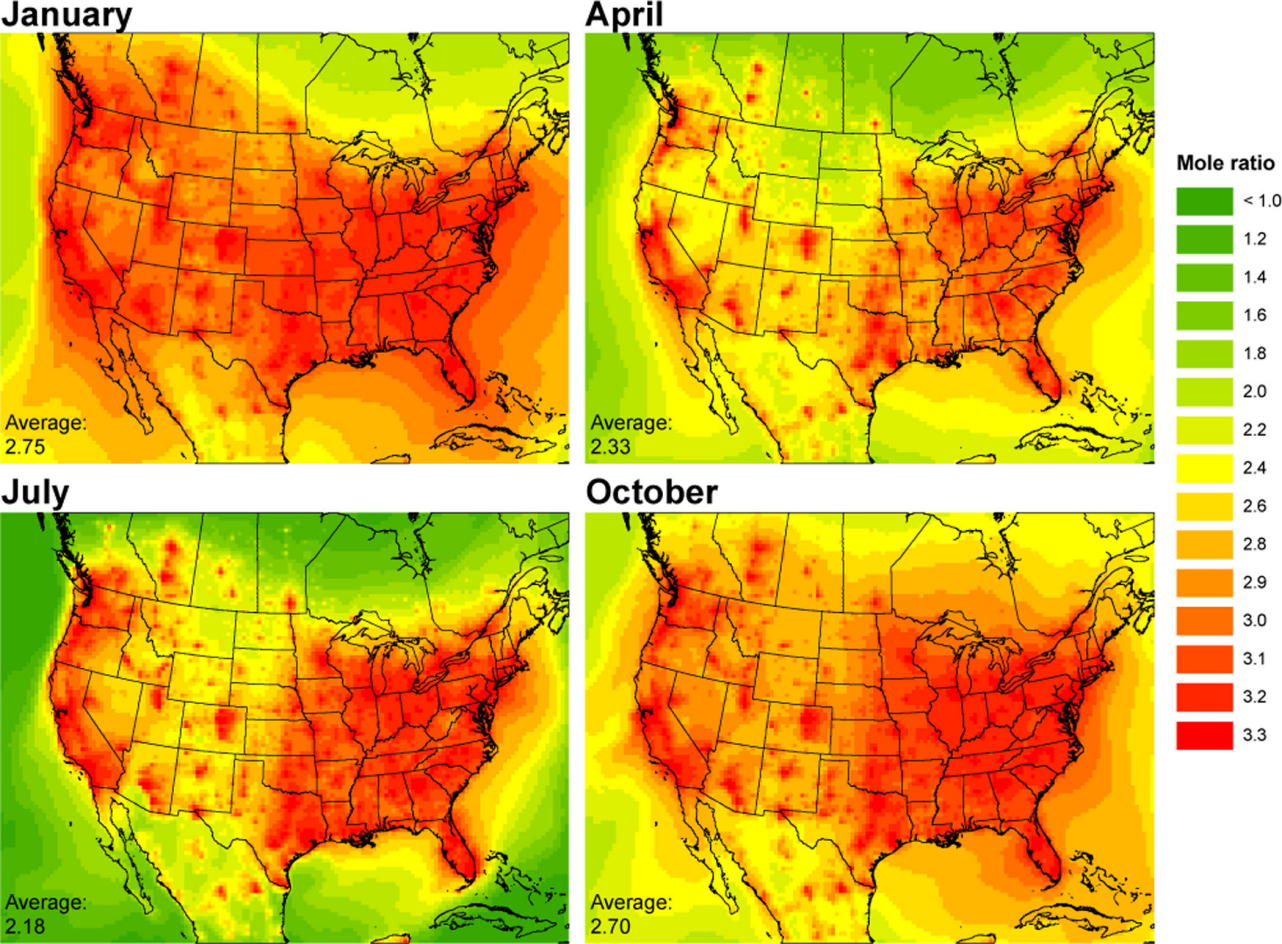
Modeled monthly averaged D_5_ /D_4_ mole ratios by season. Larger cVMS species react faster with OH. More reactive species are in the numerator; therefore, ratios decrease with air mass age.

**Figure 7.**
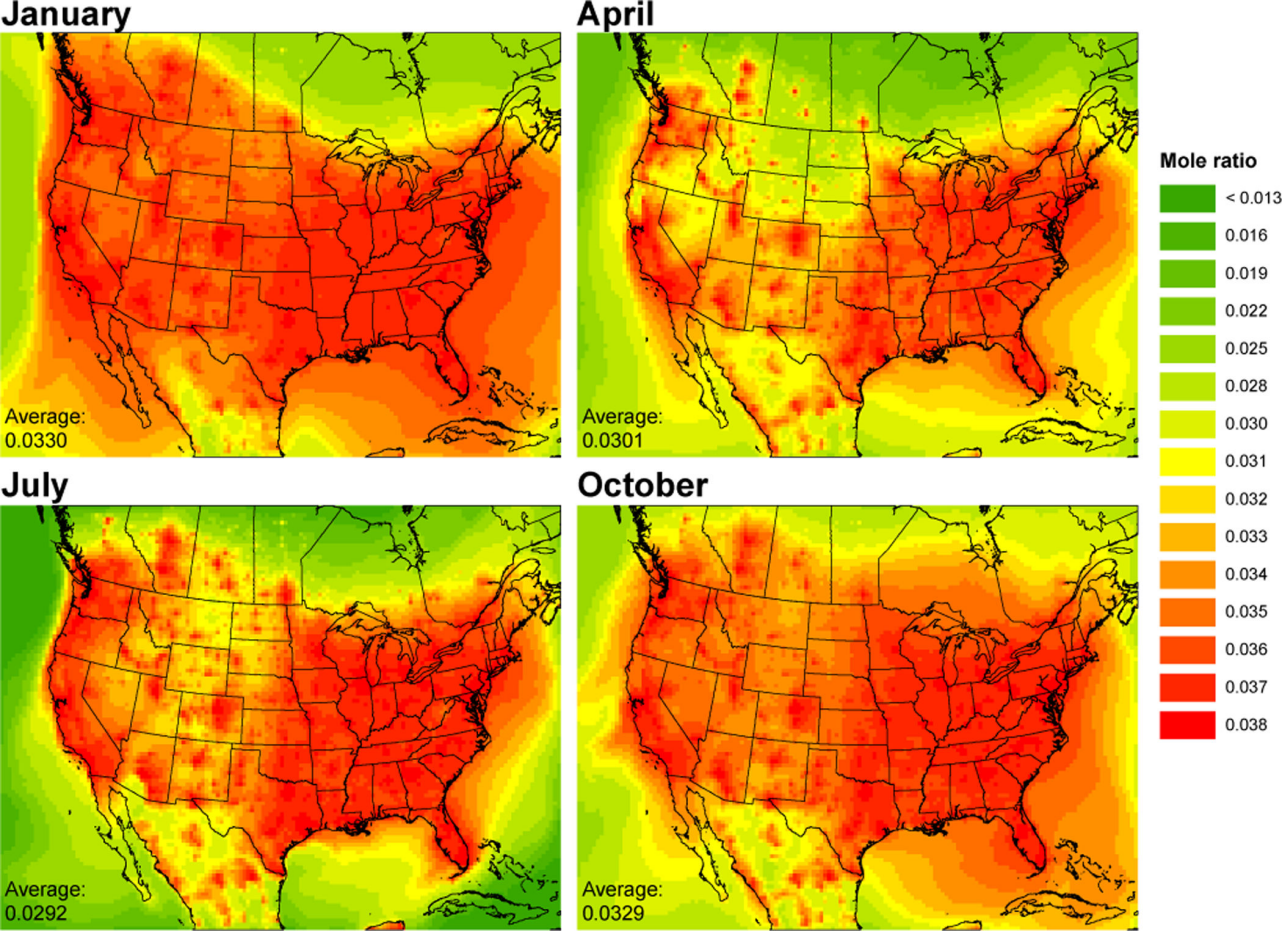
Modeled monthly averaged D_6_ /D_5_ mole ratios by season. Larger cVMS species react faster with OH. More reactive species are in the numerator; therefore, ratios decrease with air mass age.

Cyclic siloxane product ratios can be used to gain insight into emission sources and OH photochemical aging (Ahrens et al., 2014; Kierkegaard and McLachlan, 2013; Krogseth et al., 2013b, a; Yucuis et al., 2013; Navea et al., 2011). Figures 6 and 7 show the model-predicted seasonal plots of monthly averaged D_5_ /D_4_ and D_6_ /D_5_ product ratios. It is important to note that the modeling assumes D_4_ and D_6_ are emitted according to population density, at constant ratios relative to D_5_ at all locations and times. Thus, these figures emphasize the influence of differences in chemical aging. Due to differences in OH reactivity rates, cyclic siloxane reactivity increases with Si–O chain length (more methyl groups), so that D_6_ is the most reactive and D_4_ the least (Atkinson, 1991). Therefore, siloxane ratios depend on emissions, exposure to OH, and relative reactivity rates. Mole ratios are plotted with the more reactive species as the numerator; as air masses move away from emission sources and are exposed to OH, the ratio decreases due to more rapid depletion of the more reactive species. This is evident in the D_5_ /D_4_ and D_6_ /D_5_ maps, which show urban areas have the highest ratios.

Seasonal differences of the product ratios are similar for both D_5_ /D_4_ and D_6_ /D_5_ mole ratios. Urban areas exhibit almost no season-to-season difference (Table S7), as they reflect the local emission ratios. Seasonal differences are most apparent for rural and remote locations. Domain average ratios are highest in January and lowest in July which is consistent with seasonal OH fluctuations.

**Figure 8.**
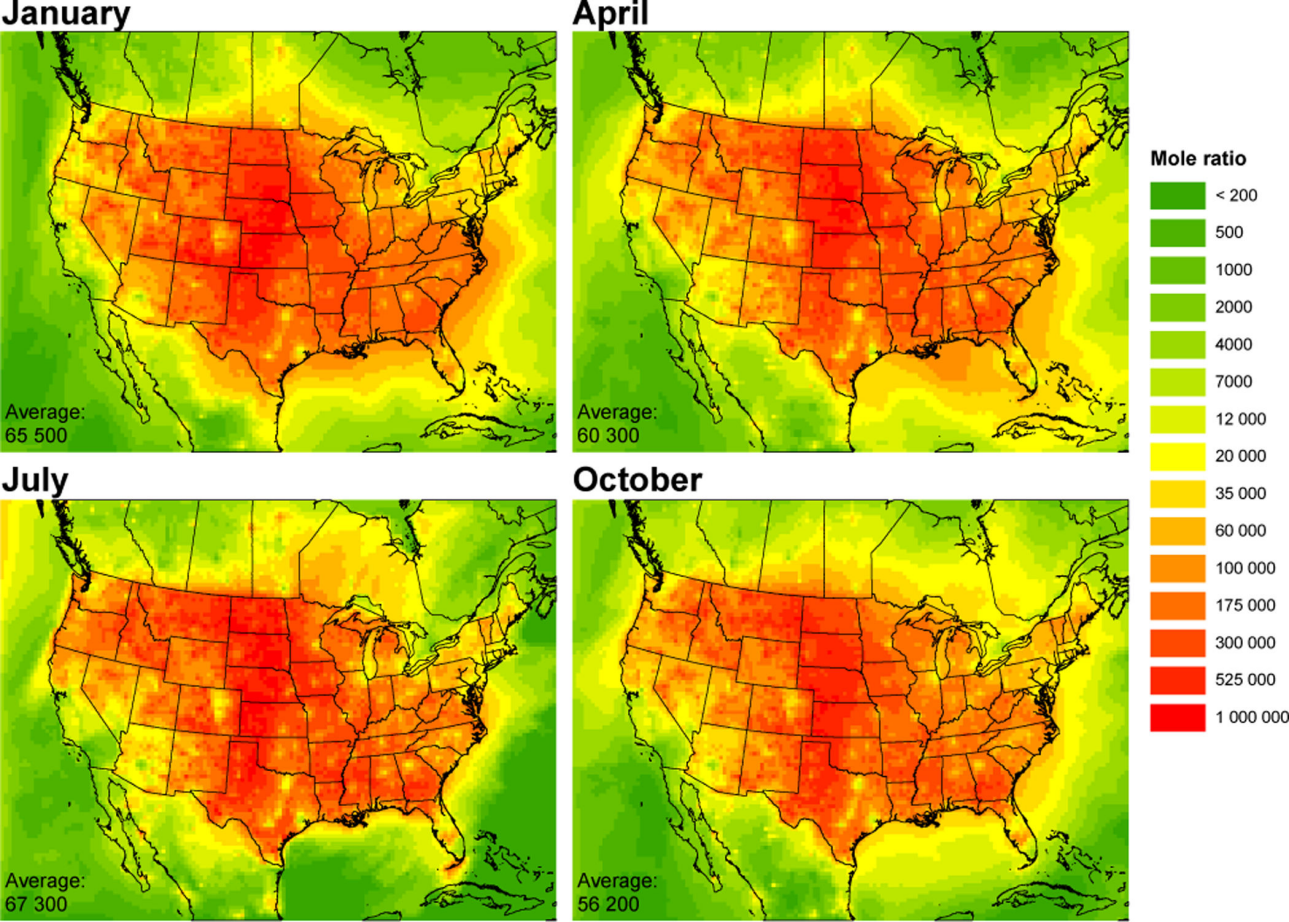
Modeled monthly averaged SO_2_ /(D_4_+D_5_+D_6_) mole ratio by season.

Since both SO_2_ and cVMS are precursors to secondary aerosol formation, and both compounds have approximately the same OH rate constant, the ratio of gas-phase SO_2_ to cVMS should predict aerosol-phase ratios of S to Si in photochemically generated particles (Bzdek et al., 2014). Figure 8 shows the seasonally modeled, monthly averaged gas-phase SO_2_ /(D_4_ +D_5_ +D_6_) mole ratios. Urban ratios exhibit lowest values which suggest photochemically generated aerosols would have increased Si composition derived from siloxane oxidation. Conversely, rural locations have high SO_2_ /cVMS ratios and expected low Si aerosol composition. This is consistent with the high nanoparticle Si measured in Pasadena, California, and Lewes, Delaware, by Bzdek et al. (2014). Seasonal variation in the SO_2_ /cVMS ratio is minor.

### 3.4 Vertical profile analysis

**Figure 9.**
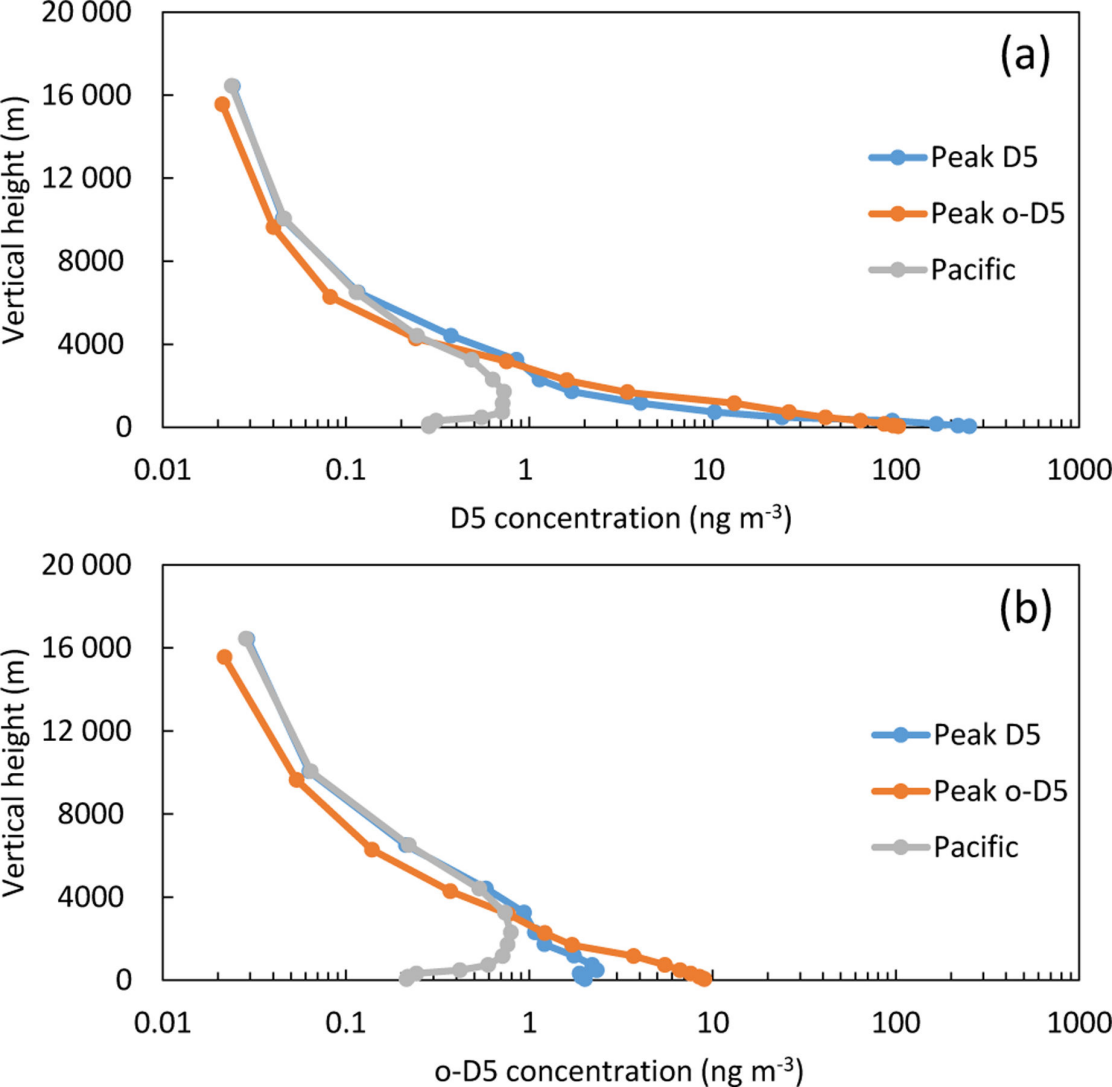
Monthly averaged vertical profiles for grid cells near Los Angeles. Plot (**a**) shows D_5_ and (**b**) o-D_5_ model concentrations. Grid cells refer to the location of maximum July D_5_, maximum July o-D_5_, and a grid cell over the Pacific Ocean.

Modeled monthly averaged D_5_ and o-D_5_ vertical profiles are shown in Fig. 9 for three grid cells near Los Angeles. The locations of the analyzed sites include the highest monthly averaged surface July D_5_ concentration, the highest averaged surface o-D_5_ concentration, and a grid cell over the Pacific Ocean. The grid cell with greatest D_5_ concentration (termed “Peak D_5_”) included cities such as Long Beach and Anaheim while the grid cell with highest o-D_5_ (“Peak o-D_5_”) was approximately 80km northeast of the peak D_5_ grid cell and included Victorville and Hesperia, California. The third location was over the Pacific Ocean (“Pacific”), approximately 195 km southwest of Los Angeles (Fig. S9).

The CMAQ model was run with 14 vertical layers; plotted is the layer top height versus the monthly averaged July D_5_ and o-D_5_ concentration. For D_5_ concentrations, both the Peak D_5_ and Peak o-D_5_ sites had highest concentrations at the surface. Over the Pacific, concentrations peaked above the surface at approximately 700–1700m. Surface D_5_ concentrations were 251, 103, and 0.3ngm^−3^ for the Peak D_5_, Peak o-D_5_, and Pacific locations, respectively. From heights 475–3000m, the Peak o-D_5_ site had higher D_5_ concentrations than the Peak D_5_ site and this is most likely due to the plume dilution from the upwind LA source. For o-D_5_ concentrations, surface concentrations were highest for the Peak o-D_5_ site (9ngm^−3^), followed by the Peak D_5_ site (2ngm^−3^), and the Pacific site (0.2ngm^−3^). From the surface to 3000m the Peak o-D_5_ grid cell had highest o-D_5_ concentrations as a result of being downwind of a major emission source and the oxidation reaction takes times to occur. Both the Peak D_5_ and Pacific sites have peak o-D_5_ concentrations not at the surface (475 and 2300m, respectively), while the “o-D_5_” site is at the surface. The low surface o-D_5_ at the peak D_5_ site could be due to low OH concentrations caused by urban OH sinks and is consistent with low modeled surface OH (Fig. S10). Vertical concentrations appear to be dependent on transport, reaction time, and OH concentrations.

## 4 Conclusions

The CMAQ model was modified to include D_4_, D_5_, D_6_, and the oxidation products to investigate urban–rural concentration gradients, seasonal variability, product and SO_2_ mole ratios, and vertical profiles. Improved model performance was observed when compared to previous modeling especially for urban areas. Concentrations are heavily dependent on population with strong urban/rural concentration gradients observed. Urban areas have highest cVMS concentrations but are not significantly influenced by seasonal variability in OH, while rural cVMS is influenced by transport and regional OH concentrations. The oxidized product concentrations are significantly lower than the parent compounds with average D_5_ concentrations up to 432ngm^−3^ and average o-D_5_ up to 9ngm^−3^. Highest oxidized siloxane concentrations occur downwind of major urban centers. Increased error for modeled D_4_ and D_6_ relative to D_5_ is hypothesized to be due to increased uncertainty in emission estimates. Future work should address these emission uncertainties by exploring seasonal, temporal, spatial, and non-personal-care product emissions.

While the parent compounds have been extensively studied, the environmental and health impact of the oxidized species have not been addressed. This is especially important since the oxidation products likely form particles. To the best of our knowledge this work provides the first estimated atmospheric loadings and spatial distribution of the oxidized species. Future work should focus on gas- and particle-phase measurements of the oxidized species to confirm particle formation in the ambient environment and to determine typical loadings in the environment. This is especially important since exposure would be expected to be highest indoors where cyclic siloxane concentrations are greatest.

## Supplementary Material

SUPPL

## Figures and Tables

**Table 1.** Monthly minimum, maximum, and average D5 and o-D5 concentrations in the lowest modeled layer for the domain.

Domain	D_5_ concentrations (ng m^−3^)				o-D_5_ concentrations (ng m^−3^)			
	January	April	July	October	January	April	July	October
Minimum	0.14	0.27	0.024	0.27	0.0031	0.037	0.0021	0.0033
Maximum	432	379	265	301	3.19	4.86	9.04	5.2
Average	6.82	5.09	4.04	6.43	0.37	0.72	0.81	0.6

**Table 2.** Toronto cyclic siloxane comparison between the CMAQ model and previous studies. Reported are the mean concentrations with ranges in parentheses.

Period	Method	Averaging period	Atmospheric concentration, mean (range)			Reference
			D_4_	D_5_	D_6_	
			(ng m^−3^)			
January	CMAQ model	24h	21.7 (5.4–45.1)	88.1 (21.5–184.8)	3.94 (0.95–8.31)	This study
April	CMAQ model	24h	20.4 (4.6–43.7)	82.1 (17.1–178.2)	3.67 (0.74–8.01)	This study
July	CMAQ model	24h	28.3 (7.5–57.0)	115.9 (30.5–233.8)	5.22 (1.37–10.54)	This study
October	CMAQ model	24h	31.0 (5.4–60.6)	126.3 (20.8–247.7)	5.67 (0.90–11.13)	This study
March 2010–April 2011	Active sampling	24h (not continuous)	16 (2.8–77)	91 (15–247)	7.3 (1.9–22)	Ahrens et al. (2014)
March 2010–April 2011	Passive sampling	∼28 days	21 (9.3–35)	140 (89–168)	11 (8.0–20)	Ahrens et al. (2014)
March 2012–June 2012	Active sampling	2–3 days	24.2 (4.7–90.9)	93.5 (22.4–355)	5.5 (1.6–17.4)	Krogseth et al. (2013b)
July 2012–October 2012	Passive sampling	80–92 days	41	122	–	Krogseth et al. (2013b)
April 2009–June 2009	Passive sampling	89 days	11	55	6.2	Genualdi et al. (2011)
April 2009–June 2009	BETR model	89 days	–	6.5	–	Genualdi et al. (2011)
April 2009–June 2009	DEHM	89 days	–	28	–	Genualdi et al. (2011)
