# Supplementary material for: Comprehensive atmospheric modeling of reactive cyclic siloxanes and their oxidation products
Source: Atmos Chem Phys. Author manuscript; Available in PMC 2019 Feb 8. (PMC6368090; doi:10.5194/acp-17-8357-2017)
Supplement: SUPPL [file NIHMS1006318-supplement-SUPPL.pdf]

Supplement of Atmos. Chem. Phys., 17, 8357–8370, 2017
https://doi.org/10.5194/acp-17-8357-2017-supplement
© Author(s) 2017. This work is distributed under
the Creative Commons Attribution 3.0 License.

Atmospheric Chemistry and Physics
Open Access
EGU

Supplement of

Comprehensive atmospheric modeling of reactive cyclic siloxanes and their oxidation products

Nathan J. Janechek et al.

Correspondence to: Charles O. Stanier (charles-stanier@uiowa.edu)

The copyright of individual parts of the supplement might differ from the CC BY 3.0 License.

Table of Contents

Section S1: Modeled Domain 2

Figure S1 - CMAQ model domain 2

Section S2: Deposition Sensitivity 3

Table S1 - Deposition sensitivity run 3

Section S3: Gridded Population Data 4

Section S4: Cyclic Siloxane Emission Rates 5

Table S2 - Cyclic siloxane emission rates 5

Section S5: Calculation of NO_x/NO_y Atmospheric Age 6

Figure S2 - Hourly NO_x and NO_y data 7

Section S6: D_4 and D_6 Boundary Concentrations 7

Figure S3 - Point Reyes OH data 8

Figure S4 - Monthly dependent D_4/D_5 and D_6/D_5 boundary ratios 9

Section S7: Analyzed Sites 10

Figure S5 - Map of sites 10

Table S3 - Site classification 11

Table S4 - Cyclic siloxane modeled surface concentrations 12

Table S5 - Oxidized cyclic siloxane modeled surface concentrations 13

Table S6 - Modeled meteorology and OH 14

Figure S6 - D_5 and o-D_5 seasonal trends 15

Table S7 - Modeled compound ratios 16

Section S8: Linear Regression 17

Table S8 - Regression results for all sites 17

Table S9 - Regression results excluding Canadian and Point Reyes sites 18

Figure S7 - PBL·WS regression fit 19

Figure S8 - OH regression fit 20

Section S9: Midwest Model Performance 21

Table S10 - Midwest model performance 21

Section S10: GAPS Model Performance 22

Table S11 - GAPS model performance 22

Section S11: Vertical Concentrations 23

Figure S9 - Analyzed grid cells 23

Figure S10 - Modeled vertical OH concentrations 24

Section S1: Modeled Domain

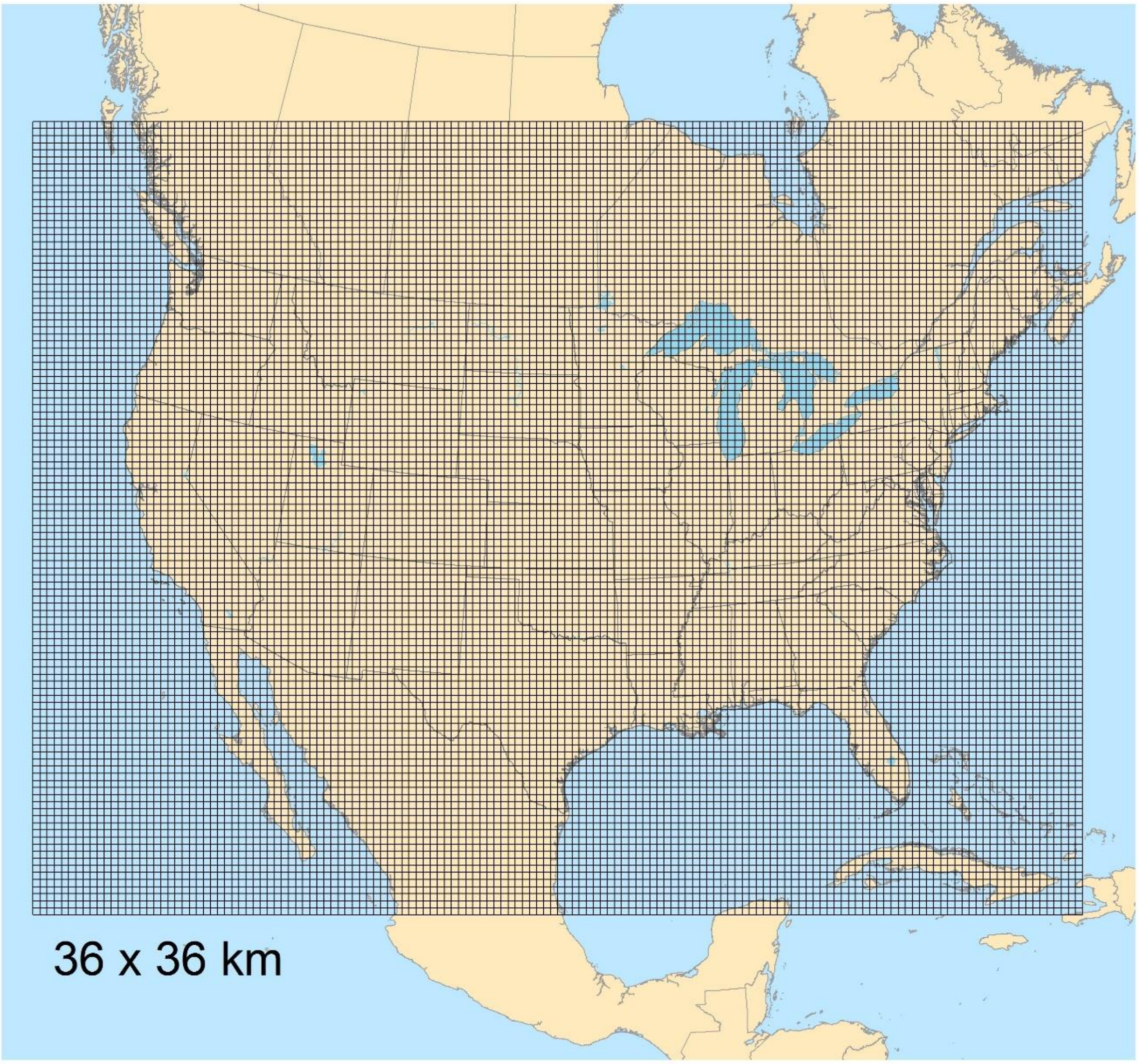

Figure S1: CMAQ model domain.

Section S2: Deposition Sensitivity

A wet and dry deposition sensitivity test compared gas phase concentrations with and without deposition as a check to verify cVMS deposition parameters. CMAQ includes treatment of dry and wet deposition (Byun et al., 1999; Roselle and Binkowski, 1999) parameterized by Henry's law coefficients, mass diffusivity, reactivity relative to HNO_3, and mesophyll resistance. However, there is considerable uncertainty in these cVMS parameterizations and only the D_4, D_5, and D_6 Henry's law coefficients (Xu and Kropscott, 2012) have been experimentally determined. A 1-week sensitivity analysis for a Chicago grid cell over the period of August 13 – August 20, 2004 was run for three scenarios 1) no deposition, 2) dry deposition only, and 3) dry and wet deposition. The addition of deposition caused gas phase concentrations to decrease, the percent change is displayed in Table S1. Wet deposition for the parent compounds was not observed but the single OH substituted oxidized species did undergo wet deposition, albeit small. Similarly, for dry deposition the parent compounds underwent less deposition than the oxidized species. Modeled wet and dry deposition agree with expected behavior based on the input parameters and the current understanding that as cVMS species are oxidized, deposition increases (Whelan et al., 2004).

Table S1: Deposition sensitivity test. August 13-20, 2004 average mixing ratios for Chicago grid cell.

	No Deposition (ppm)	Dry Deposition (ppm)	Dry and Wet Deposition (ppm)	Percent Change due to Dry Deposition (%)	Percent Change due to Wet Deposition (%)
D_4	6.16E-06	5.86E-06	5.86E-06	4.86	0.000
D_5	1.43E-05	1.36E-05	1.36E-05	4.56	0.000
D_6	1.09E-06	1.04E-06	1.04E-06	4.35	0.000
o-D_4	8.63E-08	5.89E-08	5.89E-08	31.73	0.027
o-D_5	2.85E-07	2.03E-07	2.03E-07	28.71	0.019
o-D_6	2.58E-08	1.90E-08	1.90E-08	26.43	0.014

Section S3: Gridded Population Data

Gridded population was downloaded from EPA 2011 Version 6.0 Air Emissions Modeling Platform data https://www.epa.gov/air-emissions-modeling/2011-version-60-platform. Specifically, we used population spatial surrogates. U.S. gridded population data is based on 2010 census data, Canada on 2001 census data, and Mexico from 1999/2000 census data. U.S. and Mexico population data was already gridded to the 36 km domain but Canada data was regridded from 12 km.

Section S4: Cyclic Siloxane Emission Rates

Table S2: Table of cVMS emission estimates. Numbers with superscripts correspond to [a]mean, [b]range, [c]median, and [d]maximum emission rates. Horii and Kannan (2008), Wang et al. (2009), and Dudzina et al. (2014) report exposure rate instead of air emissions. The values are likely to be similar to air emissions for Wang et al. (2009) since the products analyzed are expected to fully volatilize, the Horii and Kannan (2008) values are likely higher than air emissions since products washed down the drain are not distinguished, and the Dudzina et al. (2014) values are likely lower than air emissions since secondary volatilization from down the drain products is not included.

Location	Method	Emission Rate (mg person^{-1} day^{-1})			Reference
		D_4	D_5	D_6	
U.S. and Canada	McLachlan et al. (2010) D_5 estimate and Chicago measurements	32.8	135	6.10	*This work*
Mexico	McLachlan et al. (2010) D_5 estimate and Chicago measurements	5.92	24.4	1.10	*This work*
Berkley, CA, USA	Indoor classroom measurements	(0.048 - 30.5)[b]	(4.39 - 235)[b]	(0.46 - 7.27)[b]	*Tang et al. (2015)*
Zurich, Switzerland	Model back-calculated	-	310[a] (170-690)[b]	36[a] (19-81)[b]	*Buser et al. (2013)*
Chicago, IL, USA	Model back-calculated	-	190[a] (100-420)[b]	-	*Buser et al. (2014)*
U.S.	Dow Corning provided emissions	90	137	-	*Navea et al. (2011)*
Iowa City, IA, USA	Indoor lab measurements	(0.0090 - 0.027)[b]	(29 - 590)[b]	-	*Yucuis et al. (2013)*
Canada	Personal care product D_5 usage estimate (3,300 t/yr)	-	260	-	*Buser et al. (2014)*
U.S.	Exposure estimate from personal care products	1.08[a]	233[a]	22.2[a]	*Horii and Kannan (2008)*
Canada	Exposure estimate from lotion and antiperspirant products	98.6[d]	900[d]	-	*Wang et al. (2009)*
Netherlands and Switzerland	Exposure estimate from personal care products	0.08[c] (10.8[d])	260[c] (1,224[d])	-	*Dudzina et al. (2014)*
Portugal	Air emission estimate from personal care products	0.130[a] (0.00131 - 0.519)[b]	0.815[a] (0.00175 - 3.13)[b]	0.500[a] (0.00085 - 2.07)[b]	*Capela et al. (2016)*

Section S5: Calculation of NO_x/NO_y Atmospheric Age

D_4 and D_6 emission rates were estimated using Chicago outdoor concentrations from Yucuis et al. (2013) multiplied by the D_5 emission rate. Chicago was assumed to be representative of fresh concentrations and hence the emission ratios. Due to different OH reactivity rates, product ratios change with photochemical age. NO_x and NO_y measurements from a Chicago EPA measurement site was used to estimate the photochemical age to verify the Chicago measurements represent fresh emissions. NO_x and NO_y has been used previously to estimate photochemical age assuming NO_y is formed by $NO_2 + OH \rightarrow HNO_3$ (Slowik et al., 2011).

Hourly NO_x and NO_y data was extracted from the Northbrook, IL EPA monitoring site. Data was downloaded from Air Quality System Data Mart (http://www.epa.gov/ttn/airs/aqsdatamart). The analyzed period matched the Chicago cVMS measurements (August 13 – 21, 2011). Hourly NO_x/NO_y ratios were calculated for hours that both NO_x and NO_y measurements were available, and then averaged for the measurement period. The cVMS concentrations were calculated by averaging the primary and duplicate Chicago measurements excluding Sample 6 due to being an outlier as discussed in Yucuis et al. (2013). Several D_6 measurements were below the limit of quantification and were treated as LOQ/$\sqrt{2}$.

The photochemical age was calculated using Equation S1. The NO_x/NO_y ratio was 0.864, OH concentration $1.33\text{x}10^6$ molecules cm^{-3} interpolated for Chicago's latitude from Spivakovsky et al. (2000), and the k_{NO2} rate constant $1.08\text{x}10^{-11}$ cm^3 $mole^{-1}$ s^{-1} estimated from the JPL 2011 parameterization for 295 K and surface pressure (Sander et al., 2011). The calculated age was determined to be 2.83 hours. Plugging in this age to Equation S2, the corrected emission ratio was calculated. $[A]_0/[B]_0$ represents the photochemically corrected emission ratio, [A]/[B] the measured Chicago siloxane concentrations, *t* the photochemical age, and k_A and k_B the respective siloxane OH rate constants. Corrected D_4/D_5 and D_6/D_5 emission ratios differed by less than 1% of the measured ratios. We therefore used the measured concentration ratios without photochemical age correction.

$$t = \frac{-\ln\left(\frac{[NO]_x}{[NO]_y}\right)}{[OH]k_{NO_2}} \qquad (\boldsymbol{S1})$$

$$\left(\frac{[A]_0}{[B]_0}\right) = \frac{\left(\frac{[A]}{[B]}\right)}{e^{-[t*[OH]*(k_A-k_B)]}} \qquad (\boldsymbol{S2})$$

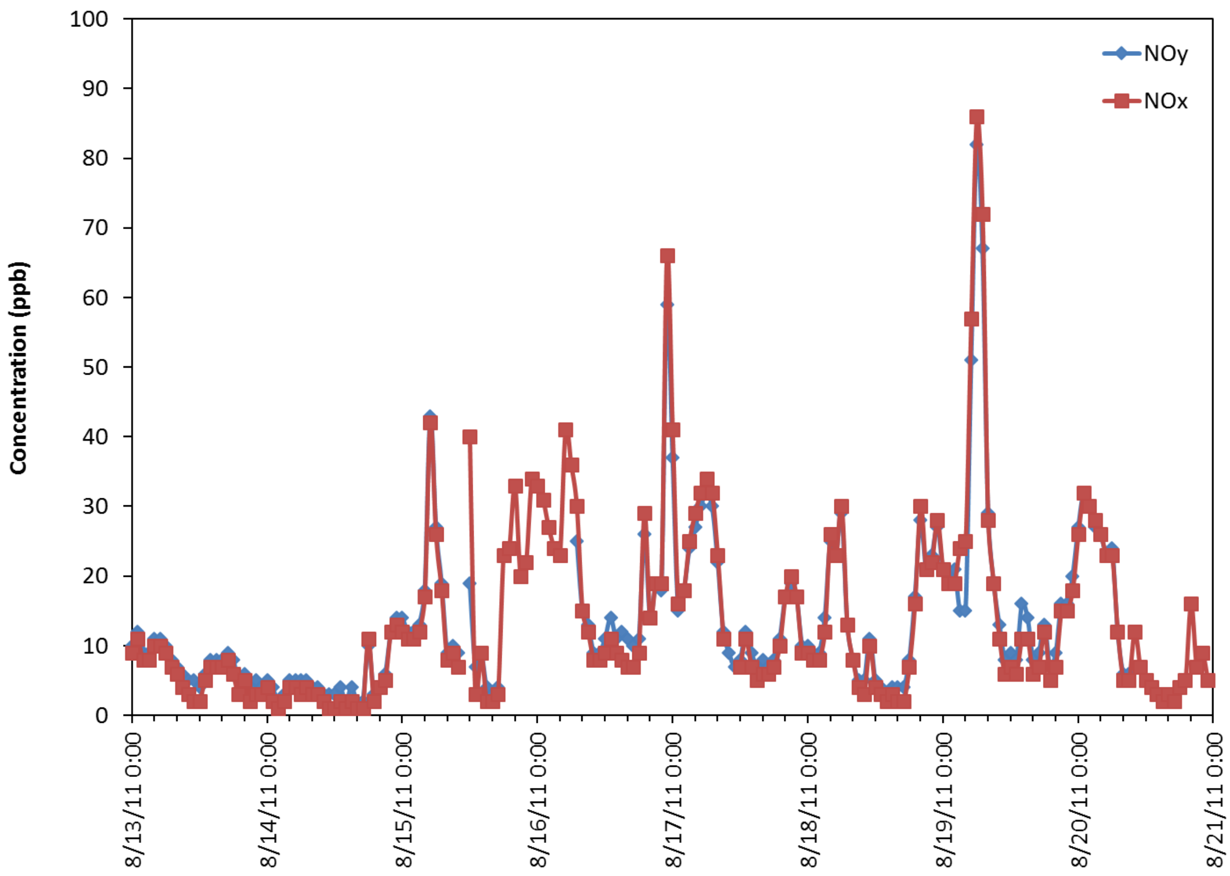

Figure S2: Northbrook hourly NO_x and NO_y measurements.

Section S6: D_4 and D_6 Boundary Concentrations

Cyclic siloxane boundary conditions were generated from the Danish Eulerian Hemispheric Model (DEHM) which simulated D_5 at 150 km resolution for the Northern Hemisphere (Hansen et al., 2008; McLachlan et al., 2010). The DEHM model provided monthly averaged, horizontally and vertically resolved D_5 concentrations. D_4 and D_6 boundary concentrations were estimated using the DEHM D_5 concentrations multiplied by an OH dependent D_4/D_5 and D_6/D_5 ratio.

Since cVMS OH rate constants vary between species, an aged background ratio will differ from a fresh emission ratio and will also be dependent on the seasonally varying OH concentrations. D_4/D_5 and D_6/D_5 boundary ratios were calculated using spring cVMS atmospheric measurements at a rural site in Point Reyes, CA (Genualdi et al., 2011). Using Equation S3, combined with the aged Point Reyes measurements and the Chicago emission measurements, we were able to calculate a photochemical age. Here *t* is the photochemical age, [A]/[B] represents the measured siloxane ratio at Point Reyes, $[A]_0/[B]_0$ the fresh emission siloxane ratio, [OH] the average OH concentration during the 3-month measurement period determined for the latitude of Point Reyes (Spivakovsky et al., 2000), and k the respective cVMS

OH rate constants. Using D_4 and D_5, a photochemical age of 17.6 days was calculated and used for all calculations.

The aged, OH dependent background D_4/D_5 and D_6/D_5 ratios were calculated using Equation S4. Here *t* is 17.6 days, [OH] the monthly dependent OH concentration as fit from Spivakovsky et al. (2000), $[A]_0/[B]_0$ the fresh emission siloxane ratio, and [A]/[B] represents the seasonally corrected boundary siloxane ratio. D_4 and D_6 concentrations were estimated by combining the seasonally specific boundary ratios with the DEHM modeled D_5. The same ratios were used for all four boundaries.

$$t = -\ln\left[\frac{\left(\frac{[A]}{[B]}\right)}{\left(\frac{[A]_0}{[B]_0}\right)}\right] * \frac{1}{[OH]*(k_A - k_B)} \qquad (\boldsymbol{S3})$$

$$\frac{[A]}{[B]} = \frac{[A]_0}{[B]_0} e^{-[OH]*t*(k_A-k_B)} \qquad (\boldsymbol{S4})$$

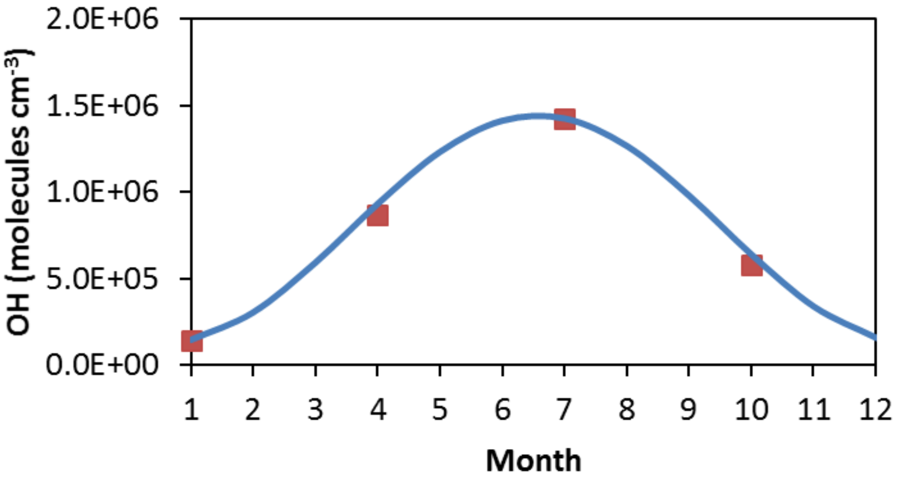

Figure S3: Spivakovsky et al. (2000) OH data for Point Reyes (38° N) latitude.

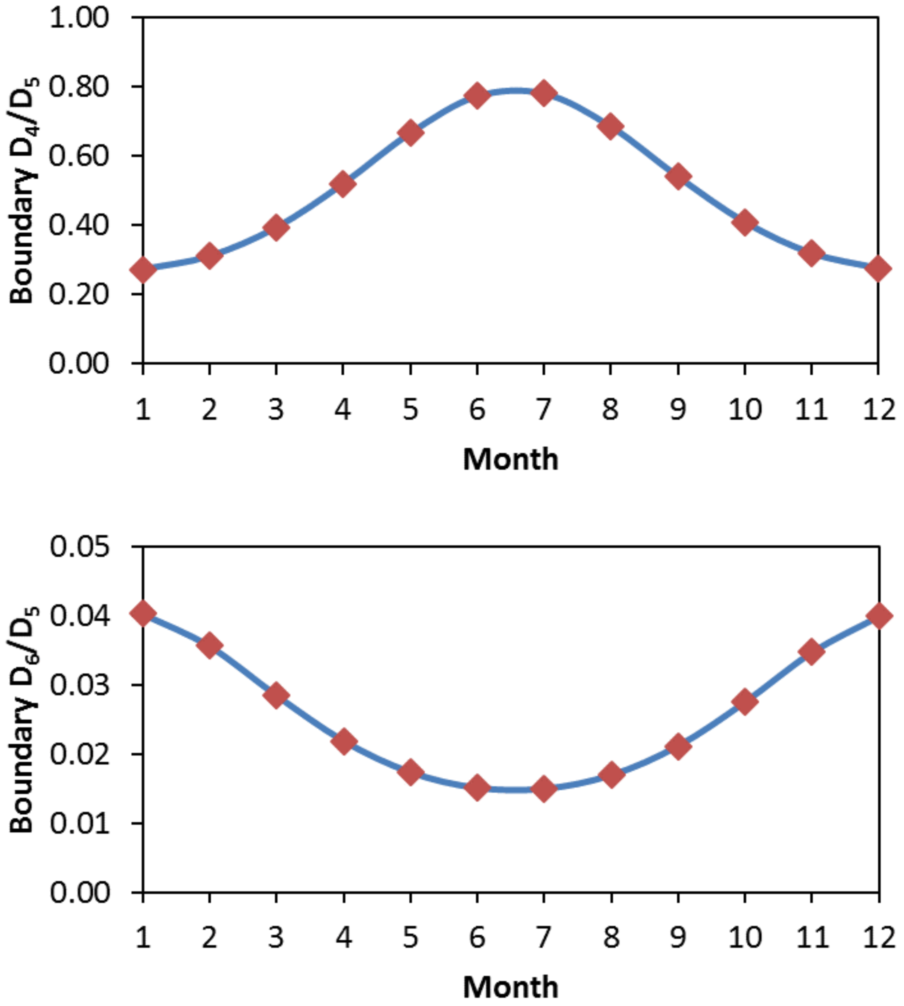

Figure S4: Monthly resolved D_4/D_5 and D_6/D_5 aged ratios.

Section S7: Analyzed Sites

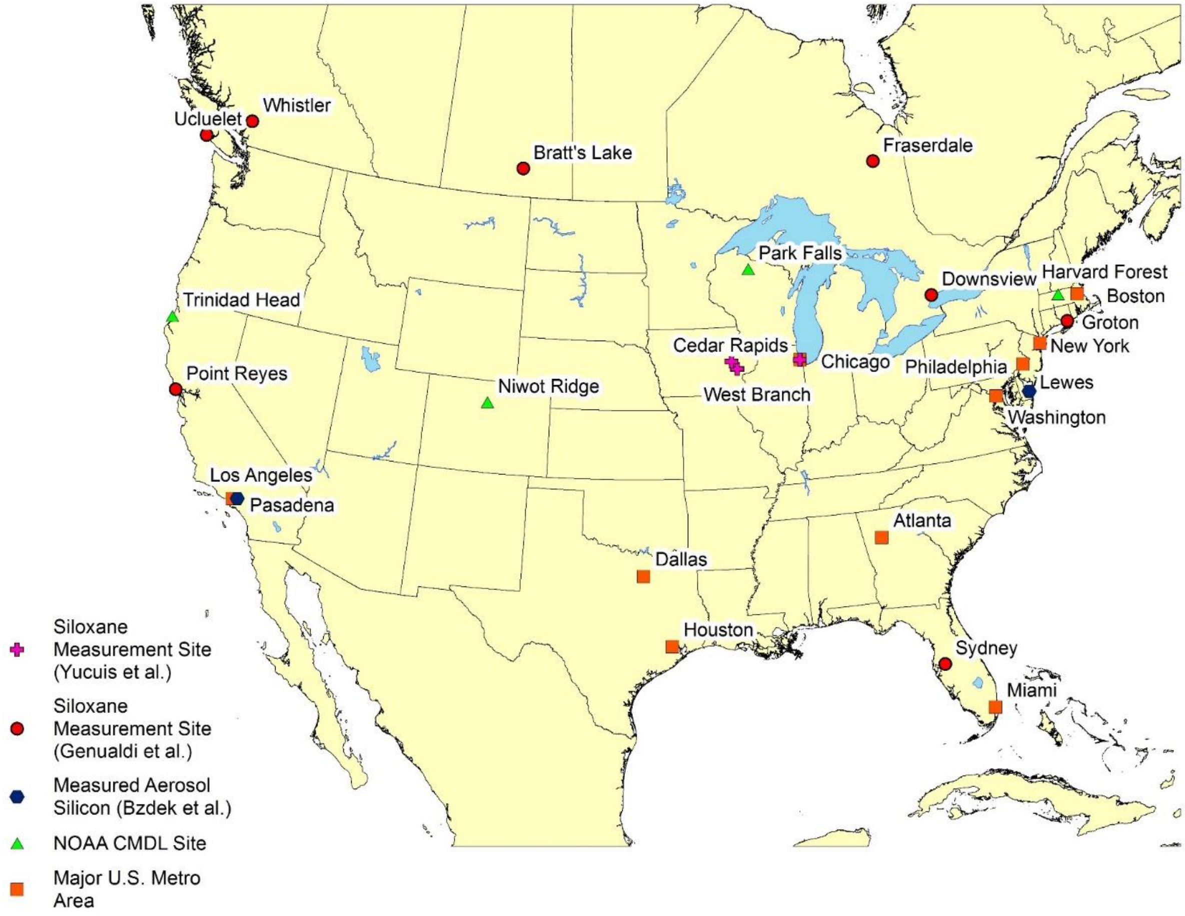

Figure S5: Analyzed 26 sites.

Table S3: Classification of 26 sites. Sites with July D_5 concentrations below 17 ng m^{-3} were classified as rural sites (in bold).

Location	Grid Cell Population	Classification	D_5 (ng m^{-3}) January	April	July	October
**Fraserdale, ON, CAN**	**12**	**Rural**	**2.88**	**1.93**	**0.76**	**5.88**
**Ucluelet, BC, CAN**	**43**	**Rural**	**6.46**	**2.46**	**0.42**	**2.42**
**Niwot Ridge, CO, USA**	**6,090**	**Rural**	**1.77**	**2.82**	**1.54**	**2.35**
**Park Falls, WI, USA**	**7,265**	**Rural**	**5.91**	**3.59**	**3.12**	**8.31**
**Trinidad Head, CA, USA**	**9,224**	**Rural**	**6.03**	**2.88**	**2.35**	**3.11**
**Whistler, BC, CAN**	**9,588**	**Rural**	**5.40**	**4.47**	**2.73**	**4.21**
**West Branch, IA, USA**	**20,291**	**Rural**	**13.24**	**8.88**	**8.82**	**18.30**
Harvard Forest, MA, USA	70,374	Urban	23.54	20.84	22.88	27.48
Lewes, DE, USA	89,714	Urban	27.62	25.59	20.79	34.00
Groton, CT, USA	100,246	Urban	29.99	43.90	44.27	30.80
**Bratt's Lake, SK, CAN**	**118,400**	**Rural**	**11.22**	**8.15**	**7.24**	**9.53**
**Point Reyes, CA, USA**	**158,892**	**Rural**	**32.64**	**16.06**	**8.38**	**18.57**
Cedar Rapids, IA, USA	193,991	Urban	19.38	15.48	17.43	23.34
Sydney, FL, USA	500,868	Urban	50.76	40.68	50.57	44.67
Dallas, TX, USA	1,191,994	Urban	83.52	63.43	52.06	93.46
Philadelphia, PA, USA	1,286,968	Urban	88.18	87.35	86.27	123.63
Atlanta, GA, USA	1,413,880	Urban	101.20	86.03	95.64	110.92
Downsview, ON, CAN	1,481,245	Urban	87.97	81.61	115.33	125.74
Boston, MA, USA	1,506,543	Urban	84.87	85.41	104.78	105.89
Miami, FL, USA	1,550,514	Urban	114.90	69.06	85.69	99.31
Washington, DC, USA	1,719,747	Urban	119.77	121.75	143.74	178.12
Houston, TX, USA	1,806,399	Urban	116.29	105.23	123.06	105.84
Pasadena, CA, USA	1,979,007	Urban	159.14	158.60	198.27	160.84
Chicago, IL, USA	2,605,915	Urban	139.34	131.79	167.80	164.02
Los Angeles, CA, USA	4,133,658	Urban	432.11	378.90	251.32	266.15
New York, NY, USA	5,245,179	Urban	234.37	227.78	265.35	301.30

Table S4: Average monthly CMAQ modeled surface cVMS concentrations (ng m^{-3}) sorted by population (highest at top of table) in analyzed grid cell. Minimum and maximum values in each column in boldface and italicized.

	D_4				D_5				D_6			
**Site**	**January**	**April**	**July**	**October**	**January**	**April**	**July**	**October**	**January**	**April**	**July**	**October**
New York, NY, USA	57.3	55.9	***64.9***	***73.6***	234	228	***265***	***301***	10.5	10.2	***11.9***	***13.5***
Los Angeles, CA, USA	***105***	***92.6***	61.3	65.1	***432***	***379***	251	266	***19.4***	***17.0***	11.3	12.0
Chicago, IL, USA	34.1	32.4	40.9	40.1	139	132	168	164	6.26	5.92	7.56	7.37
Pasadena, CA, USA	38.9	39.0	48.6	39.5	159	159	198	161	7.15	7.11	8.90	7.21
Houston, TX, USA	28.4	25.8	30.0	25.9	116	105	123	106	5.23	4.73	5.55	4.76
Washington, DC, USA	29.4	30.0	35.1	43.5	120	122	144	178	5.38	5.46	6.47	8.01
Miami, FL, USA	28.1	17.0	20.9	24.3	115	69.1	85.7	99.3	5.17	3.10	3.86	4.47
Boston, MA, USA	21.0	21.3	25.9	26.1	84.9	85.4	105	106	3.79	3.81	4.69	4.74
Downsview, ON, CAN	21.7	20.2	28.2	30.9	88.0	81.6	115	126	3.94	3.65	5.19	5.64
Atlanta, GA, USA	24.8	21.2	23.4	27.1	101	86.0	95.6	111	4.54	3.86	4.30	4.98
Philadelphia, PA, USA	21.7	21.7	21.3	30.4	88.2	87.3	86.3	124	3.95	3.90	3.86	5.54
Dallas, TX, USA	20.5	15.6	12.8	22.9	83.5	63.4	52.1	93.5	3.75	2.84	2.34	4.20
Sydney, FL, USA	12.5	10.2	12.6	11.0	50.8	40.7	50.6	44.7	2.27	1.81	2.25	2.00
Cedar Rapids, IA, USA	4.91	4.06	4.37	5.88	19.4	15.5	17.4	23.3	0.853	0.675	0.777	1.03
Point Reyes, CA, USA	8.04	4.12	2.10	4.63	32.6	16.1	8.38	18.6	1.46	0.707	0.373	0.826
Bratt's Lake, SK, CAN	2.86	2.25	1.88	2.45	11.2	8.15	7.24	9.53	0.492	0.348	0.320	0.416
Groton, CT, USA	7.62	11.2	11.3	7.91	30.0	43.9	44.3	30.8	1.32	1.93	1.95	1.34
Lewes, DE, USA	6.99	6.67	5.31	8.61	27.6	25.6	20.8	34.0	1.22	1.12	0.915	1.50
Harvard Forest, MA, USA	6.06	5.55	5.93	7.06	23.5	20.8	22.9	27.5	1.03	0.901	1.01	1.20
West Branch, IA, USA	3.42	2.46	2.28	4.66	13.2	8.88	8.82	18.3	0.576	0.378	0.389	0.804
Whistler, BC, CAN	1.39	1.30	0.728	1.11	5.40	4.47	2.73	4.21	0.235	0.185	0.118	0.181
Trinidad Head, CA, USA	1.55	0.900	0.626	0.852	6.03	2.88	2.35	3.11	0.263	0.115	0.102	0.131
Park Falls, WI, USA	1.67	1.19	0.911	2.23	5.91	3.59	3.12	8.31	0.242	0.138	0.131	0.354
Niwot Ridge, CO, USA	***0.478***	0.829	0.468	***0.649***	***1.77***	2.82	1.54	***2.35***	***0.0749***	0.116	0.0623	***0.0985***
Ucluelet, BC, CAN	1.66	***0.827***	***0.142***	0.687	6.46	2.46	***0.423***	2.42	0.282	0.0932	***0.0170***	0.0992
Fraserdale, ON, CAN	1.06	0.869	0.350	1.71	2.88	***1.93***	0.756	5.88	0.0929	***0.0559***	0.0250	0.237

Table S5: Average monthly CMAQ modeled surface oxidized cVMS concentrations (ng m^{-3}) sorted by population (highest at top of table) in analyzed grid cell. Minimum and maximum values in each column in boldface and italicized.

Site	o-D_4				o-D_5				o-D_6			
	January	April	July	October	January	April	July	October	January	April	July	October
New York, NY, USA	0.0760	0.383	***0.782***	0.404	0.454	2.11	***4.60***	2.46	0.0249	0.112	0.254	0.135
Los Angeles, CA, USA	***0.460***	***0.656***	0.315	0.576	***3.19***	***4.26***	2.01	3.89	***0.190***	***0.246***	0.114	0.228
Chicago, IL, USA	0.0622	0.278	0.427	0.219	0.359	1.32	2.30	1.27	0.0191	0.0642	0.122	0.0681
Pasadena, CA, USA	0.389	0.655	0.720	***0.666***	2.49	3.92	4.46	***4.21***	0.142	0.215	0.248	***0.237***
Houston, TX, USA	0.133	0.274	0.244	0.212	0.776	1.42	1.35	1.24	0.0417	0.0731	0.0726	0.0673
Washington, DC, USA	0.0807	0.363	0.560	0.330	0.470	1.94	3.20	2.01	0.0253	0.101	0.175	0.112
Miami, FL, USA	0.206	0.233	0.151	0.166	1.28	1.20	0.824	1.01	0.0711	0.0612	0.0439	0.0564
Boston, MA, USA	0.0579	0.246	0.555	0.239	0.334	1.28	3.14	1.43	0.0180	0.0661	0.173	0.0779
Downsview, ON, CAN	0.0424	0.210	0.373	0.162	0.246	0.994	2.08	0.948	0.0132	0.0483	0.113	0.0514
Atlanta, GA, USA	0.176	0.402	0.464	0.344	1.06	1.99	2.52	2.03	0.0582	0.0989	0.134	0.110
Philadelphia, PA, USA	0.0724	0.389	0.607	0.353	0.429	2.12	3.48	2.14	0.0234	0.112	0.191	0.118
Dallas, TX, USA	0.179	0.286	0.299	0.221	1.04	1.39	1.54	1.26	0.0554	0.0687	0.0797	0.0666
Sydney, FL, USA	0.186	0.340	0.528	0.234	1.14	1.78	3.01	1.40	0.0630	0.0914	0.163	0.0767
Cedar Rapids, IA, USA	0.0531	0.233	0.264	0.154	0.295	1.00	1.29	0.864	0.0153	0.0454	0.0659	0.0455
Point Reyes, CA, USA	0.0639	0.113	0.0639	0.0898	0.434	0.627	0.405	0.588	0.0256	0.0336	0.0237	0.0343
Bratt's Lake, SK, CAN	***0.00994***	0.104	0.0971	0.0550	***0.0547***	0.362	0.394	0.282	***0.00288***	0.0134	0.0185	0.0138
Groton, CT, USA	0.0691	0.254	0.644	0.247	0.427	1.56	4.27	1.60	0.0240	0.0900	***0.257***	0.0922
Lewes, DE, USA	0.0759	0.340	0.478	0.291	0.449	1.85	2.69	1.78	0.0245	0.0989	0.147	0.0986
Harvard Forest, MA, USA	0.0506	0.224	0.394	0.199	0.292	1.12	2.15	1.16	0.0159	0.0562	0.117	0.0626
West Branch, IA, USA	0.0535	0.238	0.269	0.165	0.298	1.03	1.31	0.933	0.0155	0.0465	0.0669	0.0494
Whistler, BC, CAN	0.0146	0.0863	0.0745	0.0306	0.0838	0.375	0.386	***0.170***	0.00453	0.0170	0.0201	***0.00889***
Trinidad Head, CA, USA	0.0246	0.0742	0.0559	0.0516	0.164	0.345	0.340	0.329	0.00966	0.0163	0.0195	0.0189
Park Falls, WI, USA	0.0213	0.125	0.172	0.104	0.114	0.486	0.778	0.582	0.00578	0.0200	0.0387	0.0306
Niwot Ridge, CO, USA	0.0288	0.128	0.219	0.0994	0.156	0.569	1.00	0.549	0.00796	0.0265	0.0494	0.0285
Ucluelet, BC, CAN	0.0138	0.0662	***0.0142***	***0.0273***	0.0955	0.288	***0.0499***	0.177	0.00587	0.0125	***0.00210***	0.0102
Fraserdale, ON, CAN	0.0172	***0.0557***	0.0732	0.0668	0.0766	***0.195***	0.266	0.367	0.00328	***0.00700***	0.0119	0.0193

Table S6: Monthly averaged modeled OH and meterology.

	OH (molec cm^{-3})				Surface Temperature (K)				Surface Pressure (Pa)				Planetary Boundary Layer Height (m)				Wind Speed (m s^{-1})			
Site	January	April	July	October	January	April	July	October	January	April	July	October	January	April	July	October	January	April	July	October
New York, NY, USA	2.15E+05	8.93E+05	2.12E+06	5.80E+05	269.3	284.2	296.4	286.5	1.01E+05	1.01E+05	1.01E+05	1.01E+05	743.5	664.6	687.2	522.0	4.18	4.17	3.23	3.20
Los Angeles, CA, USA	2.82E+05	5.38E+05	6.00E+05	4.17E+05	287.0	287.8	289.3	290.8	1.02E+05	1.01E+05	1.01E+05	1.01E+05	199.3	226.7	197.8	415.8	3.81	3.94	3.74	4.18
Chicago, IL, USA	1.81E+05	1.07E+06	2.21E+06	5.13E+05	268.0	284.5	295.3	286.3	9.93E+04	9.90E+04	9.89E+04	9.89E+04	466.3	770.1	645.7	483.3	4.81	5.09	3.54	4.20
Pasadena, CA, USA	5.35E+05	1.46E+06	1.43E+06	8.58E+05	287.1	290.2	292.6	291.1	9.98E+04	9.94E+04	9.93E+04	9.92E+04	417.2	614.6	376.5	494.8	3.14	3.17	2.77	2.91
Houston, TX, USA	4.48E+05	1.61E+06	2.45E+06	1.26E+06	287.5	293.8	300.5	298.2	1.02E+05	1.01E+05	1.01E+05	1.01E+05	466.1	627.3	549.2	561.0	4.13	4.07	3.10	4.00
Washington, DC, USA	2.46E+05	1.02E+06	2.10E+06	6.47E+05	273.1	286.5	297.6	287.6	1.01E+05	1.01E+05	1.01E+05	1.01E+05	646.5	609.1	545.4	376.2	3.48	3.69	2.70	2.53
Miami, FL, USA	1.03E+06	2.26E+06	3.04E+06	1.49E+06	292.4	295.6	300.5	298.4	1.02E+05	1.02E+05	1.01E+05	1.01E+05	473.0	738.9	488.8	495.1	3.02	3.99	2.97	3.25
Boston, MA, USA	2.29E+05	7.48E+05	1.96E+06	5.60E+05	266.4	281.8	294.6	284.7	1.01E+05	1.01E+05	1.01E+05	1.01E+05	718.9	585.0	611.2	507.6	4.34	4.09	3.07	3.46
Downsview, ON, CAN	2.13E+05	1.01E+06	1.95E+06	4.84E+05	264.2	279.9	292.7	283.7	9.81E+04	9.80E+04	9.80E+04	9.82E+04	561.0	648.7	568.3	476.8	4.43	4.58	3.23	3.81
Atlanta, GA, USA	3.75E+05	1.38E+06	2.56E+06	8.82E+05	278.9	289.5	298.9	292.5	9.84E+04	9.82E+04	9.81E+04	9.82E+04	351.3	714.6	618.2	450.2	3.75	3.65	2.96	2.94
Philadelphia, PA, USA	2.31E+05	1.01E+06	2.20E+06	5.96E+05	270.2	285.1	296.8	286.4	1.01E+05	1.01E+05	1.01E+05	1.01E+05	701.2	639.5	646.5	437.0	4.21	3.98	3.15	2.90
Dallas, TX, USA	4.56E+05	1.45E+06	3.46E+06	1.04E+06	284.2	292.4	301.6	296.1	9.96E+04	9.91E+04	9.91E+04	9.90E+04	473.8	696.3	848.3	522.2	4.52	5.17	4.57	4.08
Sydney, FL, USA	6.85E+05	1.99E+06	2.84E+06	1.41E+06	289.6	294.2	300.5	297.7	1.02E+05	1.01E+05	1.01E+05	1.01E+05	431.6	689.1	433.4	529.2	3.28	3.63	2.57	3.13
Cedar Rapids, IA, USA	2.09E+05	1.05E+06	2.22E+06	5.14E+05	267.0	285.4	295.2	285.9	9.88E+04	9.84E+04	9.83E+04	9.83E+04	413.9	720.2	523.9	457.5	5.22	4.98	3.30	4.59
Point Reyes, CA, USA	5.73E+05	1.79E+06	2.72E+06	1.19E+06	284.2	284.9	286.3	287.6	1.02E+05	1.01E+05	1.01E+05	1.01E+05	382.7	201.4	93.7	242.5	6.11	5.65	5.18	5.55
Bratt's Lake, SK, CAN	9.83E+04	1.51E+06	2.27E+06	5.37E+05	257.6	279.6	291.9	278.6	9.44E+04	9.42E+04	9.41E+04	9.39E+04	244.2	849.3	665.2	420.6	4.11	4.99	3.88	4.49
Groton, CT, USA	2.38E+05	6.82E+05	1.64E+06	5.87E+05	270.4	279.9	293.6	287.2	1.01E+05	1.01E+05	1.01E+05	1.01E+05	1023.0	151.3	103.1	686.9	9.86	5.46	4.16	6.62
Lewes, DE, USA	2.40E+05	9.40E+05	1.92E+06	6.48E+05	272.8	285.6	297.7	287.9	1.01E+05	1.01E+05	1.01E+05	1.01E+05	562.0	545.7	560.2	416.1	4.33	4.20	3.08	2.97
Harvard Forest, MA, USA	2.03E+05	7.81E+05	1.86E+06	5.17E+05	264.6	280.9	293.3	283.3	9.80E+04	9.81E+04	9.83E+04	9.85E+04	679.8	635.1	578.9	511.1	4.64	4.14	2.88	3.28
West Branch, IA, USA	2.14E+05	1.07E+06	2.20E+06	5.33E+05	267.8	285.9	295.7	286.4	9.92E+04	9.88E+04	9.87E+04	9.87E+04	420.6	748.2	531.5	455.7	5.21	5.08	3.30	4.64
Whistler, BC, CAN	2.01E+05	8.29E+05	8.97E+05	4.14E+05	270.7	278.3	287.5	278.6	8.84E+04	8.88E+04	8.90E+04	8.84E+04	237.6	590.0	650.2	343.1	3.79	3.20	3.24	3.78
Trinidad Head, CA, USA	3.63E+05	1.31E+06	2.30E+06	6.99E+05	283.9	284.9	288.4	287.8	1.02E+05	1.02E+05	1.01E+05	1.01E+05	404.9	259.7	144.2	285.6	6.21	6.14	6.24	5.25
Park Falls, WI, USA	1.61E+05	7.54E+05	1.97E+06	4.36E+05	260.7	278.3	291.3	281.7	9.61E+04	9.59E+04	9.60E+04	9.58E+04	434.1	509.3	571.7	450.4	4.54	4.08	3.43	4.53
Niwot Ridge, CO, USA	4.90E+05	1.51E+06	3.26E+06	9.85E+05	264.1	273.0	285.2	275.3	7.03E+04	7.04E+04	7.13E+04	7.06E+04	389.3	747.9	999.6	700.4	4.76	5.02	3.58	4.97
Ucluelet, BC, CAN	1.53E+05	9.79E+05	1.15E+06	4.27E+05	280.2	282.7	288.3	285.8	1.01E+05	1.02E+05	1.01E+05	1.01E+05	536.8	275.8	200.3	425.1	8.89	5.08	4.21	5.98
Fraserdale, ON, CAN	1.60E+05	5.28E+05	4.99E+05	2.44E+05	251.7	272.6	290.4	279.6	9.95E+04	9.93E+04	9.90E+04	9.90E+04	365.9	606.4	643.6	438.5	3.05	3.44	3.25	3.51

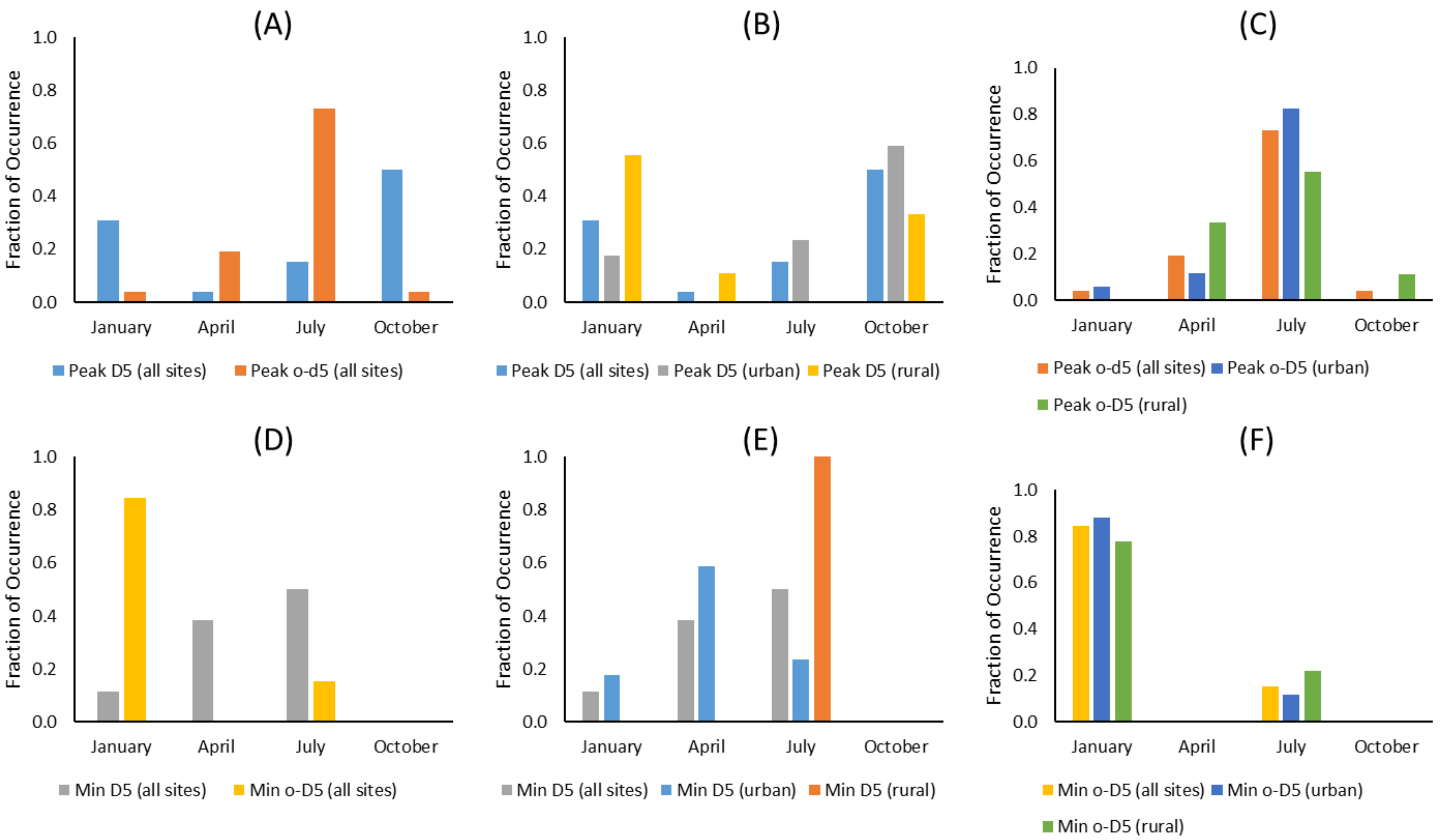

Figure S6: Seasonal trends of D_5 and o-D_5 concentrations for the 26 analyzed sites. Of the four months modeled, the month of highest or lowest average D_5 and o-D_5 concentrations are tabulated. Panel (A) shows monthly occurrence of highest D_5 and o-D_5 concentrations for all sites, (B) occurrence of highest D_5 for urban and rural sites, (C) occurrence of highest o-D_5 for urban and rural sites, (D) occurrence of lowest D_5 and o-D_5 for all sites, (E) occurrence of lowest D_5 for urban and rural sites, and (F) occurrence of lowest o-D_5 for urban and rural sites.

Table S7: Monthly averaged modeled compound ratios.

Site	D_5/D_4 January	April	July	October	D_6/D_5 January	April	July	October	SO_2/cVMS January	April	July	October
New York, NY, USA	3.27	3.26	3.27	3.27	0.0375	0.0374	0.0375	0.0375	9,294	6,937	5,960	8,247
Los Angeles, CA, USA	3.28	3.27	3.28	3.27	0.0375	0.0375	0.0375	0.0374	337	251	185	319
Chicago, IL, USA	3.27	3.26	3.28	3.27	0.0375	0.0374	0.0376	0.0375	11,538	8,616	5,662	9,615
Pasadena, CA, USA	3.27	3.26	3.26	3.26	0.0374	0.0374	0.0374	0.0374	761	283	266	503
Houston, TX, USA	3.27	3.27	3.28	3.27	0.0375	0.0375	0.0376	0.0375	9,611	4,697	4,104	5,211
Washington, DC, USA	3.26	3.25	3.27	3.27	0.0374	0.0374	0.0375	0.0375	33,544	24,260	21,360	24,316
Miami, FL, USA	3.28	3.25	3.28	3.27	0.0375	0.0374	0.0375	0.0375	5,194	4,778	1,945	2,641
Boston, MA, USA	3.24	3.21	3.23	3.24	0.0372	0.0372	0.0373	0.0373	15,531	12,303	9,211	14,497
Downsview, ON, CAN	3.24	3.23	3.27	3.26	0.0373	0.0373	0.0375	0.0374	1,546	2,973	588	2,185
Atlanta, GA, USA	3.26	3.24	3.27	3.27	0.0374	0.0374	0.0375	0.0375	30,866	28,251	18,309	22,427
Philadelphia, PA, USA	3.25	3.22	3.24	3.25	0.0373	0.0372	0.0373	0.0373	20,139	17,350	15,060	17,912
Dallas, TX, USA	3.26	3.24	3.26	3.27	0.0374	0.0374	0.0374	0.0375	23,995	15,536	15,158	11,614
Sydney, FL, USA	3.24	3.20	3.22	3.24	0.0373	0.0371	0.0372	0.0373	21,592	16,777	7,641	12,545
Cedar Rapids, IA, USA	3.16	3.05	3.19	3.18	0.0367	0.0363	0.0372	0.0368	144,645	126,766	97,014	89,098
Point Reyes, CA, USA	3.25	3.12	3.19	3.21	0.0373	0.0367	0.0371	0.0371	2,581	2,732	2,436	5,866
Bratt's Lake, SK, CAN	3.14	2.90	3.09	3.11	0.0366	0.0355	0.0368	0.0364	8,505	6,449	5,263	19,517
Groton, CT, USA	3.15	3.13	3.13	3.11	0.0366	0.0367	0.0367	0.0364	23,025	14,862	18,158	24,402
Lewes, DE, USA	3.16	3.07	3.13	3.16	0.0367	0.0364	0.0367	0.0367	49,557	39,235	40,384	34,715
Harvard Forest, MA, USA	3.11	3.01	3.09	3.12	0.0364	0.0360	0.0366	0.0364	25,561	30,775	20,004	32,611
West Branch, IA, USA	3.10	2.89	3.10	3.14	0.0363	0.0354	0.0367	0.0366	207,140	217,583	194,708	115,970
Whistler, BC, CAN	3.10	2.76	3.00	3.03	0.0363	0.0345	0.0361	0.0359	1,885	3,819	8,098	3,585
Trinidad Head, CA, USA	3.10	2.56	3.00	2.92	0.0363	0.0332	0.0361	0.0351	7,626	22,248	33,099	30,096
Park Falls, WI, USA	2.83	2.40	2.74	2.99	0.0341	0.0320	0.0350	0.0355	121,956	255,080	184,828	128,076
Niwot Ridge, CO, USA	2.96	2.72	2.62	2.90	0.0353	0.0343	0.0338	0.0349	256,457	84,118	161,838	123,540
Ucluelet, BC, CAN	3.11	2.38	2.38	2.82	0.0364	0.0316	0.0336	0.0341	3,929	3,915	2,625	7,795
Fraserdale, ON, CAN	2.18	1.78	1.73	2.75	0.0269	0.0241	0.0275	0.0336	4,935	4,603	21,266	16,309

Section S8: Linear Regression

Table S8: Linear least-squares regression results of normalized D_5 as a function of the inverse of normalized OH, PBL, and wind speed for the 26 sites. All values are dimensionless. Normalization is through division by the July value of the variable at that location.

All 26 Sites		
Sites	Variable (Coefficient); variables with $p<0.1$ bolded	Adjusted R^2
Urban	OH^{-1} (-0.0039)	-0.019
Urban	**PBL^{-1} (0.48)**	0.333
Urban	**WS^{-1} (1.35)**	0.450
Urban	**$PBL^{-1} \cdot WS^{-1}$ (0.52)**	0.492
Urban	OH^{-1} (0.0068), PBL^{-1} (0.20), **WS^{-1} (1.11)**	0.489
Urban	OH^{-1} (0.0046), PBL^{-1} (-0.32), WS^{-1} (0.37), **$PBL^{-1} \cdot WS^{-1}$ (0.68)**	0.509
Urban	OH^{-1} (-0.0083), **$PBL^{-1} \cdot WS^{-1}$ (0.52)**	0.490
Urban	PBL^{-1} (-0.31), WS^{-1} (0.29), **$PBL^{-1} \cdot WS^{-1}$ (0.70)**	0.517
Urban	**PBL^{-1} (0.24)**, **WS^{-1} (1.01)**	0.496
Urban	OH^{-1} (-0.018), **PBL^{-1} (0.52)**	0.356
Urban	**OH^{-1} (0.017), WS^{-1} (1.46)**	0.471
Rural	OH^{-1} (0.0033)	-0.040
Rural	PBL^{-1} (-1.15)	0.039
Rural	**WS^{-1} (-7.27)**	0.104
Rural	$PBL^{-1} \cdot WS^{-1}$ (-1.27)	0.035
Rural	OH^{-1} (0.062), PBL^{-1} (-1.32), **WS^{-1} (-7.00)**	0.119
Rural	OH^{-1} (-0.022), **PBL^{-1} (-18.02)**, **WS^{-1} (-23.79)**, **$PBL^{-1} \cdot WS^{-1}$ (19.69)**	0.425
Rural	OH^{-1} (0.093) $PBL^{-1} \cdot WS^{-1}$ (-1.55)	0.014
Rural	**PBL^{-1} (-17.89)**, **WS^{-1} (-23.52)**, **$PBL^{-1} \cdot WS^{-1}$ (19.47)**	0.449
Rural	PBL^{-1} (-1.14), **WS^{-1} (-7.24)**	0.148
Rural	OH^{-1} (0.10), PBL^{-1} (-1.43)	0.020
Rural	OH^{-1} (-0.028), **WS^{-1} (-7.38)**	0.069

Table S9: Linear least-squares regression results of normalized D_5 as a function of the inverse of normalized OH, PBL, and wind speed excluding Canadian and Point Reyes sites. All values are dimensionless. Normalization is through division by the July value of the variable at that location.

Without Canadian and Point Reyes Sites		
Sites	Variable (Coefficient); variables with p<0.1 bolded	Adjusted R^2
Urban	OH^{-1} (-0.0023)	-0.021
Urban	**PBL^{-1} (0.48)**	0.342
Urban	**WS^{-1} (1.31)**	0.438
Urban	**$PBL^{-1}\cdot WS^{-1}$ (0.51)**	0.493
Urban	OH^{-1} (0.0081), PBL^{-1} (0.20), **WS^{-1} (1.07)**	0.484
Urban	OH^{-1} (0.0057), PBL^{-1} (-0.29), WS^{-1} (0.38), $PBL^{-1}\cdot WS^{-1}$ (0.64)	0.501
Urban	OH^{-1} (-0.0070) **$PBL^{-1}\cdot WS^{-1}$ (0.51)**	0.487
Urban	PBL^{-1} (-0.28), WS^{-1} (0.27), $PBL^{-1}\cdot WS^{-1}$ (0.66)	0.510
Urban	**PBL^{-1} (0.25)**, **WS^{-1} (0.96)**	0.491
Urban	OH^{-1} (-0.017), **PBL^{-1} (0.51)**	0.359
Urban	**OH^{-1} (0.019), WS^{-1} (1.44)**	0.465
Rural	OH^{-1} (0.042)	-0.029
Rural	PBL^{-1} (-0.14)	-0.076
Rural	WS^{-1} (-0.0088)	-0.100
Rural	$PBL^{-1}\cdot WS^{-1}$ (-0.25)	-0.064
Rural	OH^{-1} (0.053), PBL^{-1} (-0.28), WS^{-1} (-0.29)	-0.204
Rural	OH^{-1} (0.047), PBL^{-1} (0.94), WS^{-1} (0.67), $PBL^{-1}\cdot WS^{-1}$ (-1.56)	-0.366
Rural	OH^{-1} (0.052) $PBL^{-1}\cdot WS^{-1}$ (-0.35)	-0.068
Rural	PBL^{-1} (2.37), WS^{-1} (1.66), $PBL^{-1}\cdot WS^{-1}$ (-3.34)	-0.282
Rural	PBL^{-1} (-0.21), WS^{-1} (-0.41)	-0.183
Rural	OH^{-1} (0.054), PBL^{-1} (-0.23)	-0.077
Rural	OH^{-1} (0.044), WS^{-1} (0.21)	-0.139

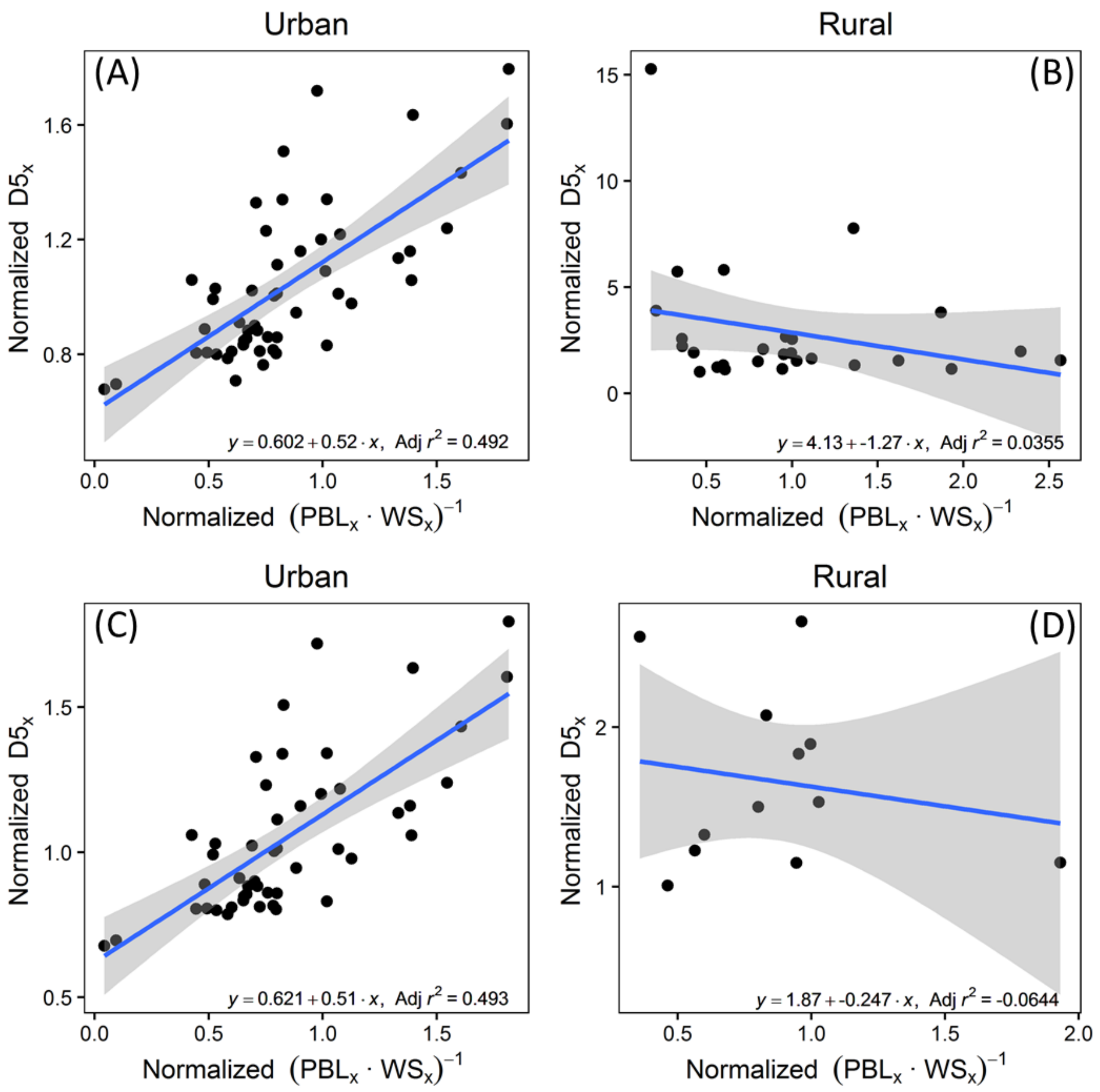

Figure S7: Linear least-squares regression fit of monthly averaged normalized D_5 concentrations versus the inverse of normalized boundary layer height and normalized wind speed for all 26 analyzed sites urban (A) and rural (B) sites. The same sites excluding Canadian and Point Reyes, CA locations are also shown for urban (C) and rural (D) sites. Normalization is through division by the July value of the quantity.

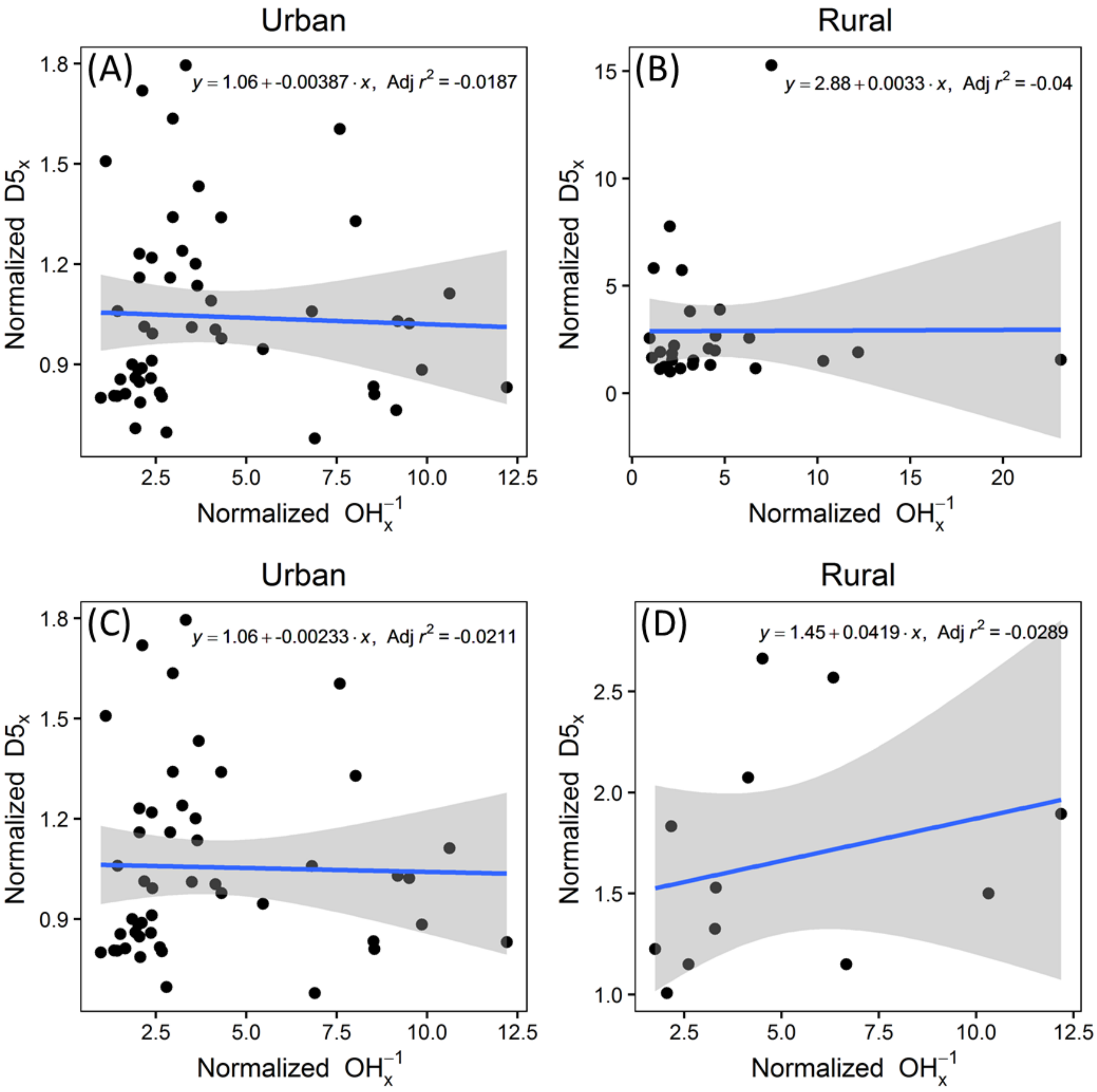

Figure S8: Linear least-squares regression fit of monthly averaged normalized D_5 concentrations versus the inverse of normalized OH concentration for all 26 analyzed sites urban (A) and rural (B) sites. The same sites excluding Canadian and Point Reyes, CA locations are also shown for urban (C) and rural (D) sites. Normalization is through division by the July value of the quantity.

Section S9: Midwest Model Performance

Table S10: Model performance to Yucuis et al. (2013) Midwest sites. Fractional bias can range from -2 to +2 while fractional error ranges from 0 to +2.

	Chicago			Cedar Rapids			West Branch		
	D_4	D_5	D_6	D_4	D_5	D_6	D_4	D_5	D_6
CMAQ Mean (ng m^{-3})	41.2	169	7.61	4.36	17.4	0.777	2.28	8.83	0.389
Yucuis et al. Mean (ng m^{-3})	56.3	232	10.1	18.9	47.9	5.56	9.94	20.1	1.65
Measured to Model Factor	1.37	1.37	1.33	4.33	2.75	7.16	4.36	2.27	4.23
Frac. Bias	-0.309	-0.313	-0.281	-1.25	-0.933	-1.51	-1.25	-0.777	-1.23
Frac. Error	0.309	0.313	0.281	1.25	0.933	1.51	1.25	0.777	1.23
Error	-15.1	-62.7	-2.49	-14.5	-30.5	-4.79	-7.66	-11.2	-1.26
% Relative Error	-26.8	-27.1	-24.6	-76.9	-63.6	-86.0	-77.1	-56.0	-76.4

$$Frac.Bias = \left(\frac{m-o}{\frac{m+o}{2}}\right)$$

$$Frac.Error = \left(\frac{|m-o|}{\frac{m+o}{2}}\right)$$

$$Error = m - o$$

$$\%\ Relative\ Error = \left(\frac{m-o}{o}\right) \times 100$$

Section S10: GAPS Model Performance

Table S11: Measurement and model fractional bias, fractional error, and absolute error. Measurement concentrations are from Genualdi et al. (2011). Fractional bias can range from -2 to +2 while fractional error can range from 0 to +2.

	CMAQ D_4			CMAQ D_5			CMAQ D_6			BETR D_5			DEHM D_5		
**Site**	**Frac. Bias**	**Frac. Error**	**Error**	**Frac. Bias**	**Frac. Error**	**Error**	**Frac. Bias**	**Frac. Error**	**Error**	**Frac. Bias**	**Frac. Error**	**Error**	**Frac. Bias**	**Frac. Error**	**Error**
Bratt's Lake, SK	-0.145	0.145	0.352	1.24	1.24	6.25	0.114	0.114	0.0376	0.383	0.383	0.900	-0.303	0.303	0.500
Whistler, BC	-1.89	1.89	43.7	-0.354	0.354	1.93	-1.56	1.56	1.31	-1.01	1.01	4.30	-0.485	0.485	2.50
Downsview, ON	0.591	0.591	9.23	0.390	0.390	26.6	-0.518	0.518	2.55	-1.58	1.58	48.5	-0.651	0.651	27.0
Fraserdale, ON	-1.45	1.45	4.53	0.0164	0.0164	0.0313	-1.52	1.52	0.354	0.417	0.417	1.00	0.100	0.100	0.200
Ucluelet, BC	-1.93	1.93	43.2	-0.992	0.992	4.84	-1.71	1.71	1.11	-	-	-	-1.07	1.07	5.10
Point Reyes, CA	-0.0195	0.0195	0.0813	0.848	0.848	9.56	0.215	0.215	0.137	-1.38	1.38	5.30	0.667	0.667	6.50
Sydney, FL	0.613	0.613	4.77	-0.674	0.674	41.3	-0.753	0.753	2.19	-1.71	1.71	75.5	-1.38	1.38	67.0
Groton, CT	0.969	0.969	7.32	-0.745	0.745	52.1	-1.45	1.45	10.1	-	-	-	-1.15	1.15	70.0
Mean	-0.407	0.950	14.1	-0.0334	0.658	17.8	-0.897	0.980	2.22	-0.812	1.08	22.6	-0.534	0.726	22.4
Median	-0.0825	0.791	6.05	-0.169	0.709	7.91	-1.10	1.10	1.21	-1.19	1.19	4.80	-0.568	0.659	5.80

$$Frac.\,Bias = \left(\frac{m-o}{\frac{m+o}{2}}\right)$$

$$Frac.\,Error = \left(\frac{|m-o|}{\frac{m+o}{2}}\right)$$

$$Error = |m-o|$$

Section S11: Vertical Concentrations

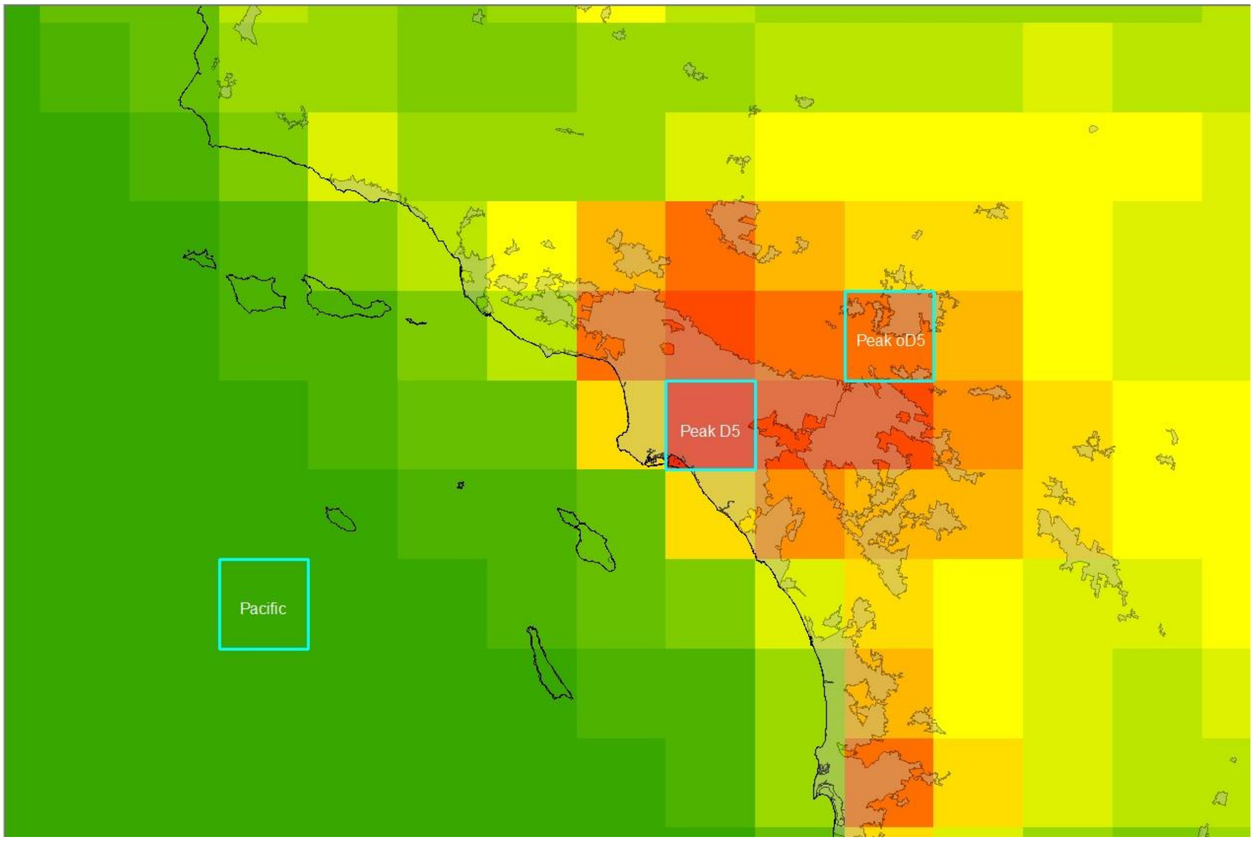

Figure S9: Grid cell locations for vertical analysis in Los Angeles area.

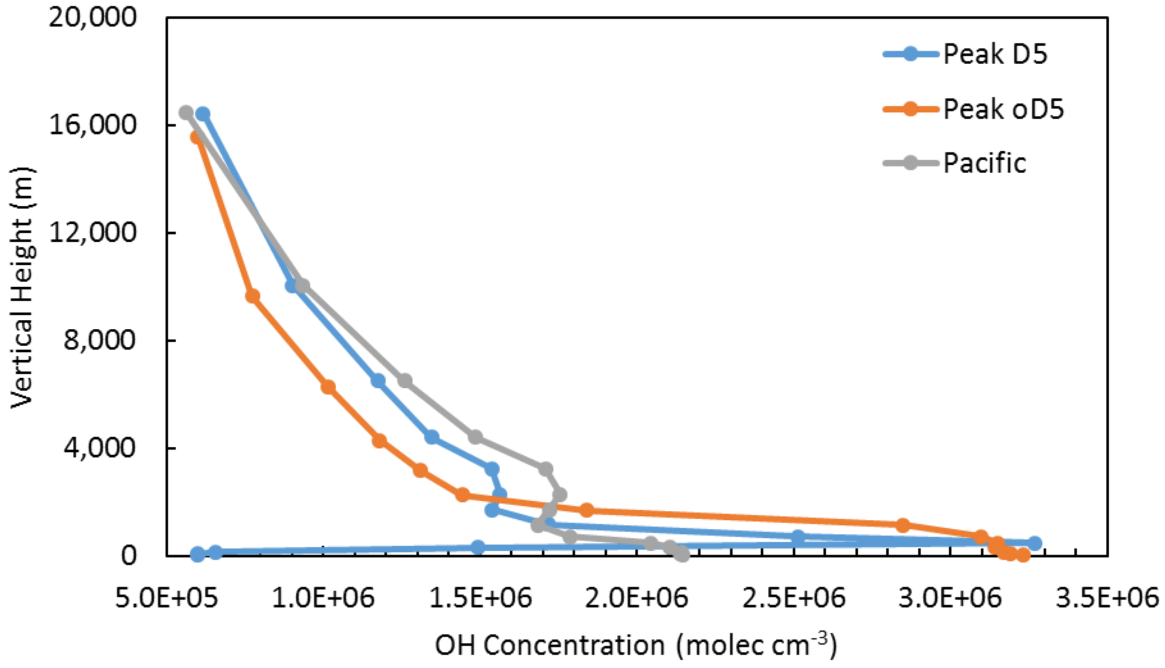

Figure S10: Monthly averaged modeled vertical OH profiles for grid cells near Los Angeles. Grid cells refer to the location of maximum July D_5, maximum July o-D_5, and a grid cell over the Pacific Ocean.

References

Buser, A. M., Kierkegaard, A., Bogdal, C., MacLeod, M., Scheringer, M., and Hungerbuhler, K.: Concentrations in Ambient Air and Emissions of Cyclic Volatile Methylsiloxanes in Zurich, Switzerland, Environ. Sci. Technol., 47, 7045-7051, doi:10.1021/es3046586, 2013.

Buser, A. M., Bogdal, C., MacLeod, M., and Scheringer, M.: Emissions of decamethylcyclopentasiloxane from Chicago, Chemosphere, 107, 473-475, doi:10.1016/j.chemosphere.2013.12.034, 2014.

Byun, D. W., Pleim, J. E., Tang, R. T., and Bourgeois, A.: Chapter 12: Meteorology-Chemistry Interface Processor (MCIP) for Models-3 Community Multiscale Air Quality (CMAQ) Modeling System, in: Science Algorithms of the EPA Models-3 Community Multiscale Air Quality (CMAQ) Modeling System, United States Environmental Protection Agency, Washington, DC, 1999.

Capela, D., Alves, A., Homem, V., and Santos, L.: From the shop to the drain - Volatile methylsiloxanes in cosmetics and personal care products, Environ. Int., 92-93, 50-62, doi:10.1016/j.envint2016.03.016, 2016.

Dudzina, T., von Goetz, N., Bogdal, C., Biesterbos, J. W. H., and Hungerbuhler, K.: Concentrations of cyclic volatile methylsiloxanes in European cosmetics and personal care products: Prerequisite for human and environmental exposure assessment, Environ. Int., 62, 86-94, doi:10.1016/j.envint.2013.10.002, 2014.

Genualdi, S., Harner, T., Cheng, Y., MacLeod, M., Hansen, K. M., van Egmond, R., Shoeib, M., and Lee, S. C.: Global Distribution of Linear and Cyclic Volatile Methyl Siloxanes in Air, Environ. Sci. Technol., 45, 3349-3354, doi:10.1021/es200301j, 2011.

Hansen, K. M., Christensen, J. H., Brandt, J., Frohn, L. M., Geels, C., Skjoth, C. A., and Li, Y.-F.: Modeling short-term variability of alpha-hexachlorocyclohexane in Northern Hemispheric air, J. Geophys. Res.-Atmos., 113, doi:10.1029/2007jd008492, 2008.

Horii, Y., and Kannan, K.: Survey of organosilicone compounds, including cyclic and linear siloxanes, in personal-care and household products, Arch. Environ. Con. Tox., 55, 701-710, doi:10.1007/s00244-008-9172-z, 2008.

McLachlan, M. S., Kierkegaard, A., Hansen, K. M., van Egmond, R., Christensen, J. H., and Skjoth, C. A.: Concentrations and Fate of Decamethylcyclopentasiloxane (D-5) in the Atmosphere, Environ. Sci. Technol., 44, 5365-5370, doi:10.1021/es100411w, 2010.

Navea, J. G., Young, M. A., Xu, S., Grassian, V. H., and Stanier, C. O.: The atmospheric lifetimes and concentrations of cyclic methylsiloxanes octamethylcyclotetrasiloxane (D(4)) and decamethylcyclopentasiloxane (D(5)) and the influence of heterogeneous uptake, Atmos. Environ., 45, 3181-3191, doi:10.1016/j.atmosenv.2011.02.038, 2011.

Roselle, S. J., and Binkowski, F. S.: Chapter 11: Cloud Dynamics and Chemistry, in: Science Algorithms of the EPA Models-3 Community Multiscale Air Quality (CMAQ) Modeling System, United States Environmental Protection Agency, Washington, DC, 1999.

Sander, S. P., Abbatt, J. P. D., Barker, J. R., Burkholder, J. B., Friedl, R. R., Golden, D. M., Huie, R. E., Kolb, C. E., Kurylo, M. J., Moortgat, G. K., Orkin, V. L., and Wine, P. H.: Chemical Kinetics and Photochemical Data for Use in Atmospheric Studies, Evaluation No. 17, Jet Propulsion Laboratory, Pasadena, CA, JPL Publication 10-6, 2011.

Slowik, J. G., Brook, J., Chang, R. Y. W., Evans, G. J., Hayden, K., Jeong, C. H., Li, S. M., Liggio, J., Liu, P. S. K., McGuire, M., Mihele, C., Sjostedt, S., Vlasenko, A., and Abbatt, J. P. D.: Photochemical processing of organic aerosol at nearby continental sites: contrast

between urban plumes and regional aerosol, Atmos. Chem. Phys., 11, 2991-3006, doi:10.5194/acp-11-2991-2011, 2011.

Spivakovsky, C. M., Logan, J. A., Montzka, S. A., Balkanski, Y. J., Foreman-Fowler, M., Jones, D. B. A., Horowitz, L. W., Fusco, A. C., Brenninkmeijer, C. A. M., Prather, M. J., Wofsy, S. C., and McElroy, M. B.: Three-dimensional climatological distribution of tropospheric OH: Update and evaluation, J. Geophys. Res.-Atmos., 105, 8931-8980, doi:10.1029/1999jd901006, 2000.

Tang, X., Misztal, P. K., Nazaroff, W. W., and Goldstein, A. H.: Siloxanes Are the Most Abundant Volatile Organic Compound Emitted from Engineering Students in a Classroom, Environ. Sci. Technol. Lett., 2, 303-307, doi:10.1021/acs.estlett.5b00256, 2015.

Wang, R., Moody, R. P., Koniecki, D., and Zhu, J.: Low molecular weight cyclic volatile methylsiloxanes in cosmetic products sold in Canada: implication for dermal exposure, Environ. Int., 35, 900-904, doi:10.1016/j.envint.2009.03.009, 2009.

Whelan, M. J., Estrada, E., and van Egmond, R.: A modelling assessment of the atmospheric fate of volatile methyl siloxanes and their reaction products, Chemosphere, 57, 1427-1437, doi:10.1016/j.chemosphere.2004.08.100, 2004.

Xu, S., and Kropscott, B.: Method for simultaneous determination of partition coefficients for cyclic volatile methylsiloxanes and dimethylsilanediol, Anal. Chem., 84, 1948-1955, doi:10.1021/ac202953t, 2012.

Yucuis, R. A., Stanier, C. O., and Hornbuckle, K. C.: Cyclic siloxanes in air, including identification of high levels in Chicago and distinct diurnal variation, Chemosphere, 92, 905-910, doi:10.1016/j.chemosphere.2013.02.051, 2013.
